# Low-dose of caffeine alleviates high altitude pulmonary edema *via* regulating mitochondrial quality control process in AT1 cells

**DOI:** 10.3389/fphar.2023.1155414

**Published:** 2023-04-04

**Authors:** Liuyang Tian, Zhilong Jia, Yan Yan, Qian Jia, Wenjie Shi, Saijia Cui, Huining Chen, Yang Han, Xiaojing Zhao, Kunlun He

**Affiliations:** ^1^ School of Medicine, Nankai University, Tianjin, China; ^2^ Medical Big Data Research Center, Medical Innovation Research Division of Chinese PLA General Hospital, Beijing, China; ^3^ National Engineering Research Center for Medical Big Data Application Technology, Chinese PLA General Hospital, Beijing, China; ^4^ Center for Artificial Intelligence in Medicine, Medical Innovation Research Division of Chinese PLA General Hospital, Beijing, China; ^5^ Research Center for Translational Medicine, Medical Innovation Research Division of Chinese PLA General Hospital, Beijing, China; ^6^ Technical Research Centre for Prevention and Control of Birth Defects, Medical Innovation Research Division of Chinese PLA General Hospital, Beijing, China

**Keywords:** caffeine, high altitude pulmonary edema (HAPE), mitochondrial, mitochondrial quality control, proteomics

## Abstract

**Backgrounds:** High-altitude pulmonary edema (HAPE) is a life-threatening disease without effective drugs. Caffeine is a small molecule compound with antioxidant biological activity used to treat respiratory distress syndrome. However, it is unclear whether caffeine plays a role in alleviating HAPE.

**Methods:** We combined a series of biological experiments and label-free quantitative proteomics analysis to detect the effect of caffeine on treating HAPE and explore its mechanism *in vivo* and *in vitro*.

**Results:** Dry and wet weight ratio and HE staining of pulmonary tissues showed that the HAPE model was constructed successfully, and caffeine relieved pulmonary edema. The proteomic results of mice lungs indicated that regulating mitochondria might be the mechanism by which caffeine reduced HAPE. We found that caffeine blocked the reduction of ATP production and oxygen consumption rate, decreased ROS accumulation, and stabilized mitochondrial membrane potential to protect AT1 cells from oxidative stress damage under hypoxia. Caffeine promoted the PINK1/parkin-dependent mitophagy and enhanced mitochondrial fission to maintain the mitochondria quality control process.

**Conclusion:** Low-dose of caffeine alleviated HAPE by promoting PINK1/parkin-dependent mitophagy and mitochondrial fission to control the mitochondria quality. Therefore, caffeine could be a potential treatment for HAPE.

## Graphical Abstract

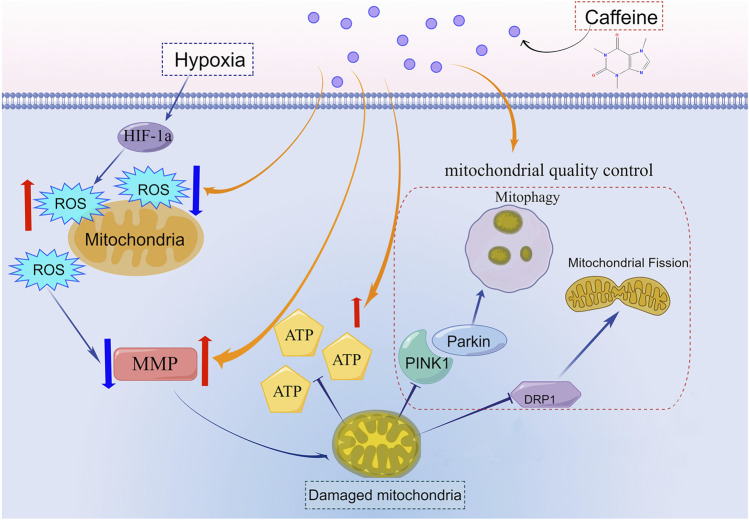

## Highlights

• Caffeine alleviated high altitude pulmonary edema *in vivo.*
• Caffeine reduced oxidative stress and stabilized mitochondrial morphology in AT1 cells under hypoxia.
• Caffeine alleviated HAPE by regulating the PINK1/Parkin-mediated mitophagy, mitochondrial fission, and mitochondrial biogenesis to maintain the quality control of mitochondria.

## 1 Introduction

Acute high altitude illness (AHAI) frequently occurs when a person ascends to a high altitude of more than 2500 m (m), which is hypoxic, hypobaric, and ultraviolet intensive (Khodaee et al., 2016). The incidence of AHAI is approximately 50%–85% for unacclimatized individuals at 4500–5500 m (Hackett et al., 1976). High altitude pulmonary edema (HAPE) is a severe subtype of AHAI, which is characterized by pulmonary edema, dyspnea, lung moist rales, cyanosis, dry cough with exertion, and pink frothy sputum (Swenson and Bartsch, 2021). The mortality rate of individuals with untreated HAPE is approximately 50% (Yanamandra et al., 2019). The typical pathogenesis of HAPE is pulmonary edema caused by excessive pulmonary vasoconstriction and abnormally high pulmonary pressures (Sharma Kandel et al., 2020). In the guidelines for prevention and treatment of acute altitude illness, acetazolamide, nifedipine, sildenafil, salmeterol, and dexamethasone are drugs used for the prevention of HAPE; acetazolamide, a diuretic, and dexamethasone are drugs used for the treatment of HAPE. However, the effectiveness of these therapies was unsatisfactory (Luks et al., 2019; Luks and Hackett, 2022). The plateau environment is a natural hypoxic environment. Hypoxia induces reactive oxygen species (ROS) accumulation and leads to oxidative stress damage in multiple diseases, such as inflammation (McGarry et al., 2018), cardiovascular disease (Giussani et al., 2014), and lung damage (Tuleta et al., 2016). Hence, inhibiting oxidative stress damage has the potential to protect the lungs from damage, holding the promise of treating HAPE.

Caffeine is a natural methylxanthine occurring in several beverages, such as coffee, tea, and cola, which is often used to fight fatigue and boost energy levels (Szlapinski et al., 2023). Caffeine has both pro-oxidative stress and anti-oxidative stress properties depending upon the dosage. Min et al. reported that intake of 10 mM caffeine causes mitochondrial dysfunction and increases oxidative stress by decreasing the level of phosphoethanolamine (PE) (Min et al., 2020). While several studies reviewed that a low dose of caffeine (10 μM) can protect several tissues from hypoxic damage by inhibiting oxidative stress damage (Ikram et al., 2020; Marte et al., 2020; Barrea et al., 2021). Such as, reducing renal fibrosis (Nilnumkhum et al., 2019), promoting neonatal neuronal survival (Li et al., 2019), and alleviating ultraviolet-induced skin senescence (Li et al., 2018).

Recent studies have revealed that caffeine is widely used to treat respiratory distress syndrome (RDS) in premature infants and improve the prognosis (Ren et al., 2015; Ines et al., 2021; Elmowafi et al., 2022). The leading cause of RDS is increased pulmonary capillary permeability caused by inflammation and endothelial dysfunction (Millar et al., 2016). The antioxidant is essential in protecting human pulmonary artery endothelial cells against excessive permeability, alleviating acute RDS (Li et al., 2020). HAPE has similar symptoms and pathological changes as RDS. However, there are no reports on whether caffeine plays a role in alleviating HAPE.

In this study, we found that a low dose of caffeine neutralized oxidative stress damage in AT1 cells by upregulating the expression of multiple genes involved in mitochondrial dynamics and mitophagy under hypoxic conditions. Caffeine promoted the PINK1/parkin-dependent mitophagy and mitochondrial fission to control the mitochondria quality both *in vivo* and *in vitro*. Moreover, we also found that pulmonary congestion and alveolar structural destruction caused by hypoxia were alleviated by caffeine in the lungs. Collectively, our results indicated that caffeine could be a potential treatment for HAPE.

## 2 Materials and methods

### 2.1 Animal care

Eight-week-old male C57BL/6 mice were purchased from SPF (Beijing) Biotechnology (Beijing, China). Animals were fed under conditions of constant humidity (50% ± 5%), temperature (23°C ± 2°C), and illumination (12 h light/dark cycles). All animal experiments were conducted following the National Institutes of Health’s Guide for the Care and Use of Laboratory Animals (NIH publication No. 80–23, revised in 1996). The Animal Ethics Committee of the Chinese PLA General Hospital (SQ2020030) approved all experimental procedures involving animals.

### 2.2 Mice and caffeine treatment

Mice were divided into three groups randomly (*n* = 6 in each) which received: (1) Control group (Con); (2) Mice were exposed to hypobaric hypoxic conditions for 3 days (Hypoxia group, Hypo); To construct the mice models of high altitude pulmonary edema, we exposed mice to a hypobaric hypoxic chamber for 3 days, which referred to our previous studies and reported methods (Ni et al., 2014; Tian et al., 2021). (3) Mice were exposed to hypobaric hypoxic conditions 3 days, and given caffeine intervention by oral gavage caffeinated water (Velazquez et al., 2020; Szlapinski et al., 2023) (Caffeine from Sigma–Aldrich, St. Louis, MO, U.S., 0.2 g/kg/d, dose volume of 10 mL/kg/d body weight) (Hypoxia + caffeine group, Hypo + Caf). The human equivalent dose based on body surface area (Km value for humans = 37 and for mice weighing 25 g = 3) was 2.5 mg/kg/day (Nair and Jacob, 2016), which was approximately 1-2 cups of canned coffee or specialty espresso (McGuire, 2014). The hypobaric hypoxic environment was constructed to simulate a 5,500-m-high atmospheric environment using a FLYDWC50-1C hypobaric hypoxic cabin (Guizhou Fenglei Air Ordnance LTD., Guizhou, China).

### 2.3 HE staining

The lung specimens were fixed in 4% paraformaldehyde overnight, embedded in paraffin, and sectioned into 6 μm thick slices. Tissue sections were stained with hematoxylin for 5 min and eosin for 3 min. HE-stained sections were analyzed using an optical microscope (Nikon, Japan).

### 2.4 Dry and wet weight ratio of lung

The pulmonary dry and wet weight ratio reveals the severity of pulmonary edema. To calculate the dry/wet ratio, we weighed the whole left lung at the initial removal and after drying it in an oven at a temperature of 160°C for 72 h.

### 2.5 LC-MS analysis

Liquid chromatography-tandem mass spectrometry (LC-MS) was carried out as described previously (Goldman et al., 2019). Briefly, cells or lungs were sonicated three times on ice using a high-intensity ultrasonic processor (Scientz, China) in the lysis buffer. Then the proteins were digested by trypsin. The peptides were subjected to NSI source followed by tandem mass spectrometry (MS) in Q Exactive TM Plus (ThermoFisher Scientific, U.S.) coupled online to the ultra-performance liquid chromatography (UPLC). The proteomic experiments of lungs were finished by the ptm-biolab (Hangzhou, China). The proteomic experiments of AT1 cells were completed by Metware Biotechnology Co. (Wuhan, China). The resulting LC-MS data were processed using the Maxquant search engine (v.1.5.2.8). The minimum score for modified peptides was set at> 40, and FDR was <1%. The LFQ (label-free quantitation) intensity was mean-based scaled per protein in all the samples, which was used in the downstream analysis.

### 2.6 Differential expression analysis

The analysis of differentially expressed proteins (DEPs) was implemented through the Limma package in the R language. The DEPs are defined as |logFC|>1.5 and *p*-value < 0.05. Proteins with logFC >1.5 are defined as upregulated proteins. Proteins with LogFC < −1.5 are defined as downregulated proteins.

### 2.7 GO annotation

Gene Ontology (GO) is a major bioinformatics initiative to unify the representation of genes and gene product attributes across all species (Ashburner et al., 2000). Gene Ontology (GO) annotation proteome was derived from the UniProt-GOA database (http://www.ebi.ac.uk/GOA/). Proteins were classified by Gene Ontology annotation based on three categories: biological process, cellular component, and molecular function.

### 2.8 KEGG pathway annotation

The Kyoto Encyclopedia of Genes and Genomes (KEGG) database were used to annotate protein pathways (Kanehisa et al., 2021). Firstly, using KEGG online service tool KAAS to annotate the protein’s KEGG database description and then mapping the annotation result on the KEGG pathway database using KEGG online service tool KEGG mapper.

### 2.9 Enrichment of pathway analysis

The KEGG database was used to identify enriched pathways by a two-tailed Fisher’s exact test to test the enrichment of the DEPs against all identified proteins. The pathway with a *p*-value <0.05 was considered significant.

### 2.10 Protein-protein interaction network

All DEPs were searched against the STRING database version 11.5 for protein-protein interactions (Szklarczyk et al., 2021). We fetched all interactions that had a confidence score ≥0.7 (high confidence) and visualized the interaction network using Cytoscape String App (Doncheva et al., 2019). The significant modules with a score ≥5 were screened out *via* MCODE.

### 2.11 The construction of the cell model

Type Ⅰ alveolar epithelial (AT1) cells were purchased from Beijing Qianzhao Xinye Biology Science and Technology Company (Beijing, China). Cells were cultured in DMEM with 10% Fetal bovine serum (FBS) and 1% antibiotics (Penicillin and Streptomycin). The hypoxic model cells were constructed by exposure to 1% O_2_ for 24 h, according to the pathological hypoxic cell model construction (McKeown, 2014).

### 2.12 Cell activity measurements

AT1 cells were seeded in the 96-well plate at 8000 cells/100 ul/well. The CCK-8 solution (Cell Counting Kit-8, Coolaber, Beijing) was added to each well according to the manufacturer’s instructions. Plates were incubated at 37°C and measured the absorbance at 450 nm using a microplate reader (BioTek, U.S.). The concentration of 0 μM, 5 μM, 10 μM, 15 μM, 20 μM, 40 μM, and 80 μM caffeine were co-cultured with cells, and the optimum concentration was calculated.

### 2.13 Mitochondrial real-time ATP rate assay

AT1 cells were inoculated in a 24-well cell culture plate and incubated at 37°C with 5% CO_2_ overnight. The following compounds were injected into the cell culture medium: oligomycin (1.5 μM), a mixture of rotenone (0.5 μM) and antimycin A (0.5 μM). The oxygen consumption rate (OCR) and extracellular acidification rate (ECAR) were measured by Seahorse XFe/XF24 Analyzers (Agilent Technologies, U.S.) according to instructions.

### 2.14 Mitochondrial Mito Stress Test kit assay

AT1 Cells were inoculated in a 24-well cell culture plate and incubated at 37°C with 5% CO_2_ overnight. The following compounds were injected into the cell culture medium: oligomycin (1.0 μM), FCCP (1.0 μM), and a mix of rotenone and antimycin A (0.5 μM)) to measure ATP production, maximal respiration, and non-mitochondrial respiration, respectively.

### 2.15 Mitochondrial glycolysis rate assay

AT1 Cells were inoculated in a 24-well cell culture plate and incubated at 37°C with 5% CO_2_ overnight. The following compounds were injected into the cell culture medium: a mixture of rotenone (0.5 μM) and antimycin A (0.5 μM), and 2-deoxy-D-glucose (2-DG, 500 mM). The proton efflux rate (PER) and glycolytic rate were measured by Seahorse XFe/XF24 Analyzers (Agilent Technologies, U.S.) according to instructions.

### 2.16 Determination of reactive oxygen species (ROS) in the cytoplasm and mitochondria

The CellROX™ Deep Red reagent is a novel fluorogenic probe for measuring cellular oxidative stress which is to be fluorescent upon oxidation by reactive oxygen species. The cytoplasmic ROS was measured using CellROX^®^ Deep Red Reagent according to the manufacturer’s instructions (CellROX^®^ Oxidative Stress Reagents (C10422), ThermoFisher Scientific, U.S.). To detect the mitochondrial superoxide, we used the MitoSOX which could selectively detect the superoxide in the mitochondria of live cells (Kalyanaraman et al., 2012; Forman et al., 2015) (MitoSOX™ Red mitochondrial superoxide indicator (M36008), ThermoFisher Scientific, U.S.). Briefly, cells were incubated with the CellROX^®^/MitoSOX Reagent at a final concentration of 5/10 μM for 30 min at 37°C, then removed medium and washed cells three times with PBS. The cells were supplemented with an antifade mounting medium with DAPI. Images were acquired using the OLYMPUS FV1000 inverted confocal microscope (Japan).

### 2.17 Determination of mitochondrial membrane potential

The mitochondrial membrane potential (MMP) was measured using JC-1 (5′,6,6′-tetrachloro-1,1′,3,3′-tetraethylbenzimidazolylcarbocyanine iodide), which exhibits potential-dependent accumulation in mitochondria according to the manufacture instruction (MitoProbe™ JC-1 Assay Kit (M34152), ThermoFisher Scientific, U.S.). The JC-1 dye loading solution was diluted into a final concentration of 2 μM. Then, the cells were co-incubated with JC-1 dye at 37°C with 5% CO2 for 30 min. Then the medium was removed and cells were washed three times with PBS. The cells were supplemented with an antifade mounting medium with DAPI. Images were acquired using the OLYMPUS FV1000 inverted confocal microscope (Japan).

### 2.18 Immunofluorescence

For confocal immunofluorescence analysis, cells were fixed in 4% formaldehyde for 10 min, then permeated in 0.2% Triton X-100 for 5 min and blocked in 3% BSA for 2 h. The incubation condition was as follows: CoraLite^®^488-conjugated TOM20 Monoclonal antibody (CL488-66777, Proteintech, China, 1:200 dilution), CoraLite^®^594-conjugated LAMP1 Monoclonal antibody (CL594-67300, Proteintech, China, 1:200 dilution), MTCO2 Mouse Monoclonal Antibody (A-6404, ThermoFisher Scientific, U.S., 1:200 dilution) and Parkin Polyclonal Antibody (PA5-13399, ThermoFisher Scientific, U.S., 1:50 dilution) at 4°C over-night. After being washed three times with TBST, the cells were co-incubated with/without fluor-conjugated goat anti-rabbit secondary antibody (red) (SA00013-4, Proteintech, China, 1:200 dilution) and fluor-conjugated goat anti-mouse secondary antibody (green) (SA00013-1, Proteintech, China, 1:200 dilution). The cells were supplemented with an antifade mounting medium with DAPI. Images were acquired using the OLYMPUS FV1000 inverted confocal microscope (Japan). Mitophagy was measured by the co-expression fluorescence of yellow dots (LAMP1 and Tom20 overlay) per field.

### 2.19 Transmission electron microscopy

The freshly harvested cells were processed as described previously (Labuschagne et al., 2019). The sections with a thickness of 70 nm were observed under an HT7800 transmission electron microscope (Hitachi, HT7800).

### 2.20 Isolation of mitochondria

Mitochondria were isolated by a mitochondrial isolation kit (Mitochondrial extraction Kit (SM0020), Beijing Solarbio Science & Technology Company, China). Briefly, the AT1 cells were washed three times with PBS and collected after centrifugation at 800 *g* for 10 min 500 μL lysis buffer was added to resuspend the cells. Then the cells were ground 30–40 times in a 0°C ice bath in a small-capacity glass homogenizer. Cell debris and nuclei were removed by centrifugation at 1000 *g* for 5 min, 4°C. And mitochondrial fractions were collected by centrifugation at 12,000 g for 15 min, 4°C.

### 2.21 Quantitative real-time PCR

AT1 cells were collected and washed 3 times with PBS. Trizol reagents were added to dissociate nucleoproteins (TRIzol™ (15596026), ThermoFisher Scientific, U.S.). Total RNA was extracted following the manufacturer’s protocol. Then the total RNA was reversed into cDNA (GoTaq^®^ qPCR Master Mix (A6001), Promega, U.S.). Quantitative RT-PCR analysis was actualized by Bio-Rad CFX96 Real-Time PCR Detection System (Bio-Rad, U.S.). The sequences of primers used in this study are listed in Supplementary Table S3.

### 2.22 Western blotting

The protein lysates were prepared in RIPA buffer and phenylmethylsulfonyl fluoride (Merck Millipore, U.S.). The concentration of protein was measured by the BCA assay (NO.23225, ThermoFisher Scientific, U.S.). The quality of 20 μg proteins was loaded per condition. Proteins were separated by 4%–20% SDS-polyacrylamide gel electrophoresis and then transferred to nitrocellulose membranes. The membranes were incubated in 5% milk for 2 h before incubation with primary antibodies at 4°C overnight. Primary antibodies were as follows: Platelet-type phosphofructokinase (PFKP, 8164, 1:1000 dilution), Pyruvate kinasem1/2 (PKM1/2, 3106, 1:1000 dilution), Tom20 (42406, 1:1000 dilution), LC3A/B (4108, 1:1000 dilution), PINK1(4946, 1:1000 dilution), Parkin (L211, 1:1000 dilution), Bnip3L (12396, 1:1000 dilution) and FUNDC1 (49240, 1:1000 dilution) were purchased from Cell Signaling Technology (U.S.). DRP1 (ab56788, 1:1000 dilution) and Hexokinase II (ab209847, 1:1000 dilution), ACTIN (ab6276, 1:1000 dilution) were purchased from Abcam (U.S.). Glucose-6-phosphate isomerase protein (GPI, PA5-97517) and P-Ser65-Parkin (Ser65) (p-Parkin, PA5-114616) were purchased from ThermoFisher Scientific (U.S.), Tim23 (67535-1-Ig, 1:4000 dilution), PGC1α (66369-1-Ig, 1:10000 dilution) were purchased from Proteintech (China). After being washed three times with TBST, the membranes were incubated with corresponding secondary antibodies (1:5000 dilution) and visualized using the instrument Amersham imager 600 (GE Healthcare Life Sciences, U.S.). The relative levels of individual proteins to control ACTB/VDAC were analyzed by ImageJ software (Madison, WI, U.S.).

### 2.23 Statistical analysis

Results were represented by means ± S.D. Statistical analyses were performed using GraphPad Prism 8.0 Software (GraphPad Software Inc., America). The choice of statistical tests for pairwise comparisons was made based on whether the data satisfies a normal distribution and/or equal variance tests. For normally distributed continuous variables, an independent sample *t*-test was used for statistical significance between two experimental groups. Otherwise, a Mann-Whitney U test was deployed. *p*-value (*p*) < 0.05 was considered statistically significant.

## 3 Results

### 3.1 Caffeine alleviates HAPE might by regulating mitochondrial OXPHOS

**FIGURE 1:**
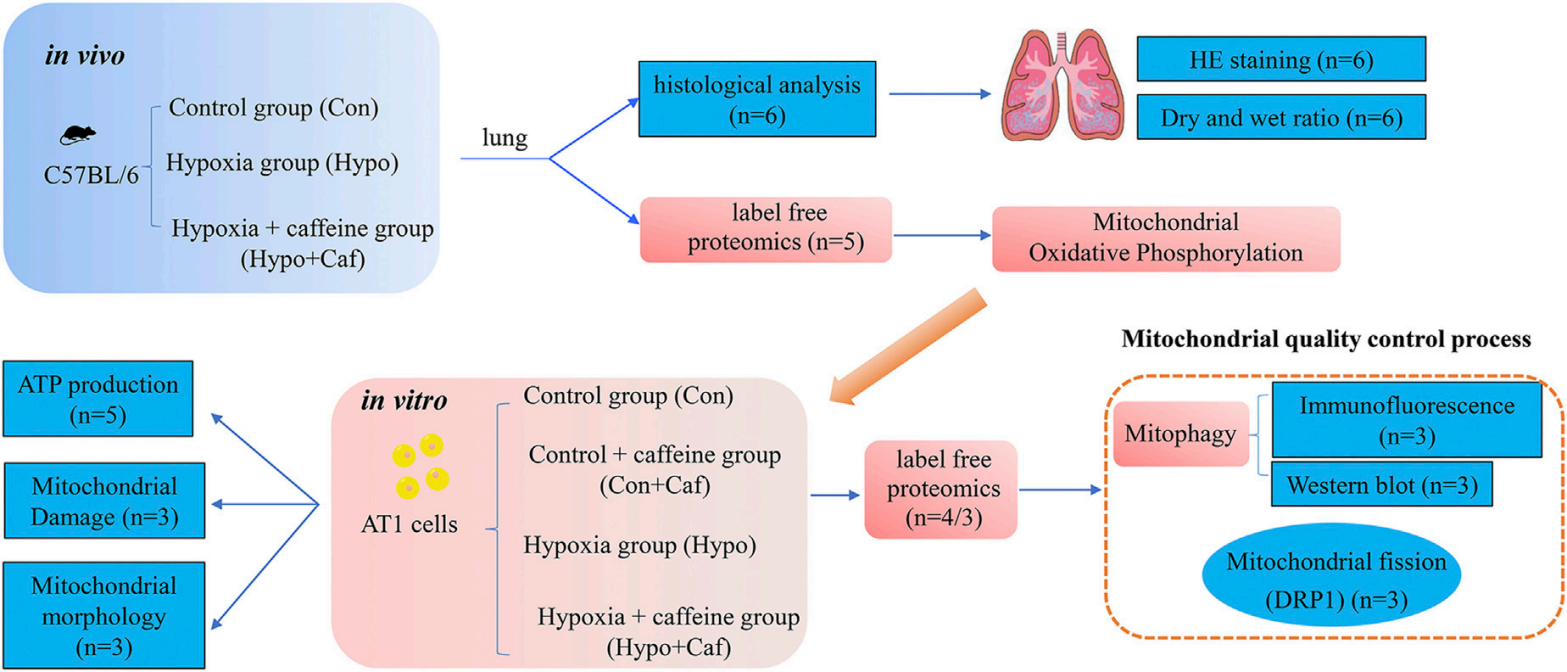
The schematic overview of the experimental workflow. We designed three groups, consisting of control, hypoxia and hypoxia + caffeine groups for *in vivo* mice experiments. Via histological and proteomics analysis of pulmonary tissues, we tested the function of caffeine for HAPE at the tissue level and discovered mitochondria probably involved in preventing HAPE. Then, we designed four groups (control group, control + caffeine group, hypoxia and hypoxia + caffeine group) in the *in vitro* AT1 cell-based experiments to detail the function of mitochondria in HAPE *via* mitochondrial oxidative phosphorylation, ROS synthesis, mitochondrial membrane potential, mitochondrial morphology, mitochondrial fission and proteomics analysis, identifying key mitophagy proteins and pathway.

**FIGURE 2:**
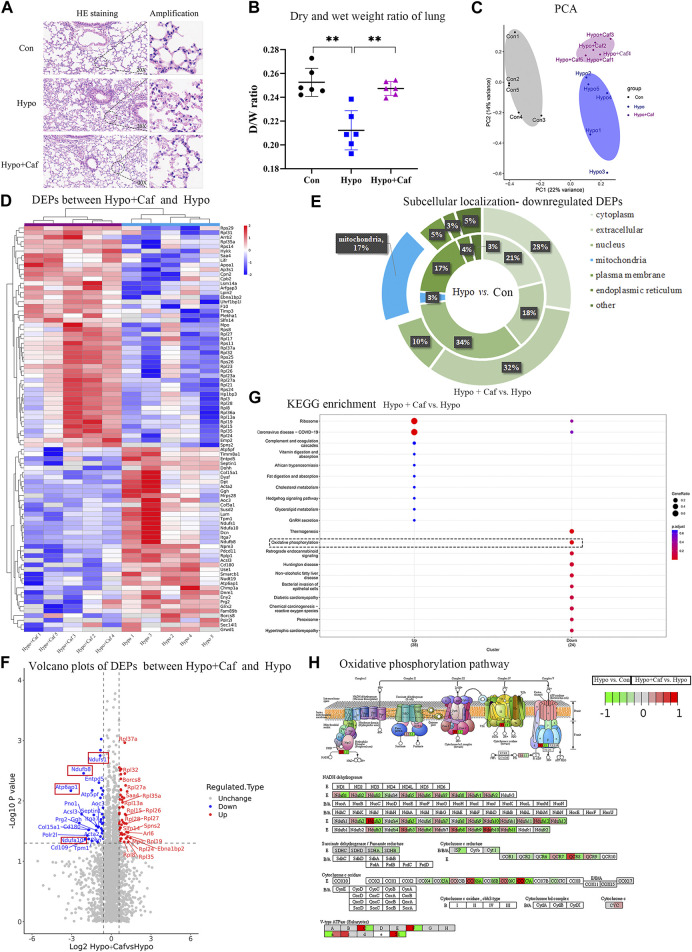
Caffeine alleviates HAPE by acting on mitochondria. **(A)** Images of hematoxylin and eosin–stained lung tissues. Hypoxia led to pulmonary congestion and alveolar structural destruction, but caffeine suppressed these damages. **(B)** Dry and wet weight ratio of lung. The dry and wet weight ratio of lung dramatically declines in Hypo group, suggesting the successful mice model of pulmonary edema model. Caffeine neutralized the decrease in dry and wet weight ratio. **(C)** Principal component analysis of 15 samples based on the expression of all the proteins. These proteins could clearly separate the three groups from each other. **(D)** Heatmap of DEPs between Hypo and Hypo + Caf groups. The hierarchical clustering showed a clear separation of the two groups. **(E)** Subcellular localization of downregulated proteins. The internal ring refers to the subcellular localization ratio of DEPS between Hypo and Con group, while the external ring refers to the subcellular localization ratio of DEPS between Hypo + Caf and Hypo group. 3% of proteins localized to mitochondria (shown in blue) were downregulated in hypoxia. 17% of proteins localized to mitochondria were downregulated by caffeine in hypoxic condition. **(F)** Volcano map of DEPs between Hypo group and Hypo + Caf group. The top 20 DEPs were labeled and contained multiple mitochondrial subunits (red box), such as Ndufs1, Ndufa10, Ndufb8 and Atp6ap1. **(G)** KEGG pathway enrichment of DEPs between Hypo group and Hypo + Caf group. The most significantly downregulated pathway by caffeine was oxidative phosphorylation (dashed box). **(H)** The DEPs in oxidative phosphorylation pathway. Hypoxia upregulated the expression of proteins in the oxidative phosphorylation pathway (the left side of the box), and the addition of caffeine decreased the expression of these proteins (the right side of the box) to maintain their normal levels. **p < 0.01.

To examine the effect of caffeine on alleviating HAPE, we conducted multi-level biological assays and bioinformatic analysis *in vivo and in vitro*. The schematic overview of our experimental workflow was shown in Figure 1. We found that pulmonary congestion and alveolar structural destruction were severe after hypobaric hypoxia intervention, but caffeine attenuated these changes (Figure 2A). Compared with the control group, the dry and wet weight ratio (D/W weight ratio) significantly declined in the Hypo group (16% reduction, *p* = 0.0001, Figure 2B), suggesting severe pulmonary edema. Notably, caffeine neutralized the decrease in the dry and wet weight ratio (16% increase, *p* = 0.0004, Figure 2B) and relieved pulmonary edema. To explore the mechanism of caffeine in alleviating HAPE, we performed label-free quantitative proteomics for the mice lungs to search for crucial proteins. We detected 64,300 unique peptides from 3,105,702 MS spectra (Supplementary Figure S1A). Principal component analysis (PCA) with all the identified proteins could be separated between the Con group, Hypo group, and Hypo + Caf group (Figure 2C). The PCA results also validated that our model construction was successful. We found 327 DEPs between Hypo and Control groups and 93 DEPs between Hypo + Caf and Hypo groups (Supplementary Figure S1B; Supplementary Table S1). The heatmap with hierarchical clustering of DEPs between the Hypo + Caf group and the Hypo group showed a clear separation (Figure 2D). The subcellular location of these DEPs varied greatly, and mitochondria were the organelle with the greatest difference. 16% of upregulated DEPs in the Hypo group compared with the Con group were located in the mitochondria (Supplementary Figure S1C), and 17% of downregulated DEPs in the Hypo + Caf group compared with the Hypo group were located in the mitochondria (Figure 2E). Volcano plots with the labeled top 20 DEPs were shown (Figure 2F; Supplementary Figure S1D). Notably, we found multi-subunit genes of mitochondria, such as Ndufs1, Ndufa10, Ndufb8, and Atp6ap1 (Figure 2F). Gene Ontology (GO) enrichment analysis revealed that the downregulated DEPs between Hypo + Caf and Hypo groups were significantly enriched in cellular respiration, mitochondrial respiratory complexes and NADH dehydrogenase oxidative phosphorylation (Figure 2H; Supplementary Figure S1E–G). The enriched KEGG pathways of DEPs between the Hypo and Hypo + Caf groups showed that the most significantly downregulated pathway was oxidative phosphorylation (Figure 2G). In detail, hypoxia upregulated the expression of oxidative phosphorylation pathway-associated proteins, and caffeine maintained its normal levels (Figure 2H). The mRNA expressions of the mitochondrial oxidative phosphorylation genes were decreased by caffeine in hypoxia (Supplementary Figure S2B). Hence, we concluded that the mechanism of caffeine alleviating HAPE may be through the regulation of the mitochondrial oxidative phosphorylation pathway.

### 3.2 Effects of caffeine on mitochondrial ATP metabolism

Mitochondria are the powerhouses of animal cells. To explore the effects of caffeine on mitochondrial bioenergetics, we measured energy metabolism phenotypes using AT1 cells, which cover more than 95% of the internal surface of the lung (Eaton et al., 2009). One of the functions of AT1 cells is the reabsorption of excess lining fluid from the alveolar surface (Baloglu et al., 2020). The effect of cell viability was detected by the CCK8 assay. Hypoxia decreased cell viability, and caffeine increased cell viability in a dose-dependent manner. We found that caffeine at 10–20 μM significantly increased the cell viability of AT1 cells, and 15 μM caffeine was the optimum concentration (Supplementary Figure S2A).

**FIGURE 3:**
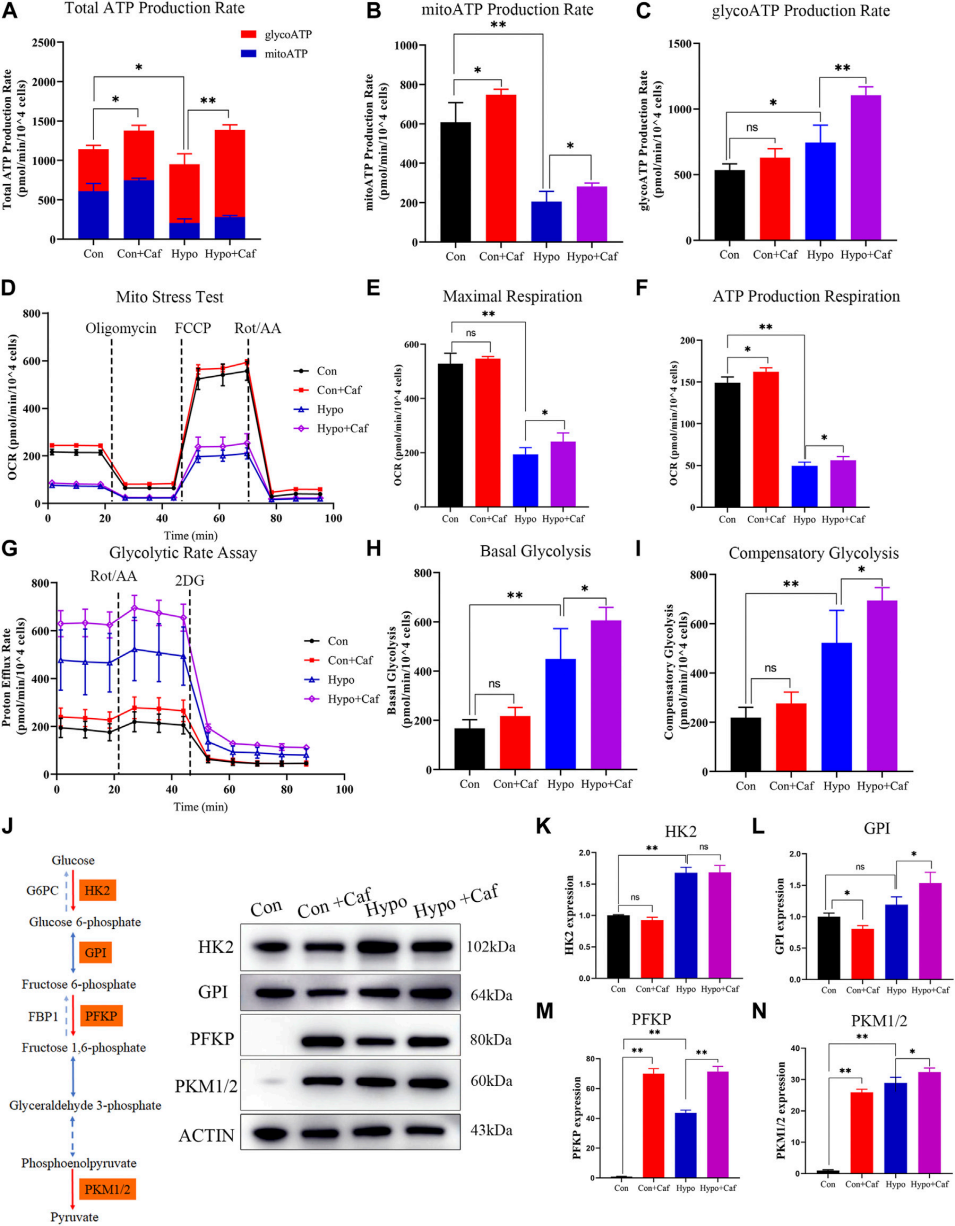
Effects of caffeine on mitochondrial ATP production. **(A–C)** The total ATP produced by mitochondrial oxidative phosphorylation and glycolysis. Caffeine increased the total ATP production in normoxia (Con + Caf group vs. Con group) and hypoxia (Hypo + Caf group vs. Hypo group). **(B)** Rate of ATP production produced by mitochondrial oxidative phosphorylation. The mitoATP production rate dramatically decreased in hypoxia, and caffeine can mitigate the decrease in the Hypo + Caf group. **(C)** Rate of ATP production produced by glycolysis. We found a significant increase in glycoATP production rate in hypoxia, and caffeine significantly amplified this change. **(D)** The OCR was measured using Seahorse XF Cell Mito Stress Test. **(E)** Maximal respiration and **(F)** ATP production respiration of the Mito Stress Test was calculated. Hypoxia decreased the maximal and ATP production respiration, and caffeine reversed these changes. **(G)** The rate of cellular glycolysis. By measuring the proton efflux rate, we calculated the rate of glycolysis. **(H)** The basal glycolysis and **(I)** the compensatory glycolysis of mitochondria were calculated. Hypoxia caused the accumulation of basal and compensatory glycolysis, and caffeine significantly amplified these changes. **(J–N)** The representative Western blot images and summarized data of rate-limiting enzymes in glycolysis. ACTB was used as an internal reference. *p < 0.05, **p < 0.01.

The total ATP production was composed of mitochondrial oxidative phosphorylation-produced ATP (mitoATP) and glycolysis-produced ATP (glycoATP). The mitoATP and glycoATP were calculated by measuring the oxygen consumption rate (OCR) and extracellular acidification rate (ECAR), separately (Supplementary Figures S2C,D). The total ATP was decreased in hypoxia, but reversed by caffeine (Figure 3A). The mitoATP dramatically decreased in hypoxia, and caffeine mitigated the decrease in the Hypo + Caf group (Figure 3B). The glycoATP was significantly increased in hypoxia, and caffeine amplified this change (Figure 3C). Cell Mito Stress Test was detected to explore the mitochondrial activity (Figure 3D). Hypoxia decreased basal respiration, but caffeine did not affect it (Supplementary Figure S2E). The maximal and ATP production respiration decreased in hypoxia, and caffeine reversed these changes (Figures 3E,F). GlycoATP production is almost four times of mitoATP in hypoxia (Figure 3A). As a result, glycolysis becomes the primary source of ATP synthesis in hypoxic conditions. To determine the rate of glycolysis, we measured the glycolytic rate and maximum glycolytic capacity using Seahorse XF Glycolytic Rate Assay (Figure 3G). The basal glycolysis and the compensatory glycolysis were increased in hypoxia compared to the control, and caffeine further amplified the increase (Figures 3H,I). The expressions of rate-limiting enzymes in glycolysis were detected by Western blot (Figure 3J). Compared with normoxia, hypoxia increased the expression of HK2, GPI, PFKP, and PKM1/2, and caffeine amplified the increased degree of PFKP and PKM1/2 (Figures 3K–N). These findings suggest that caffeine increased the glycolytic capacity to supply energy in hypoxia.

### 3.3 Caffeine reduces mitochondrial damage triggered by hypoxia

**FIGURE 4:**
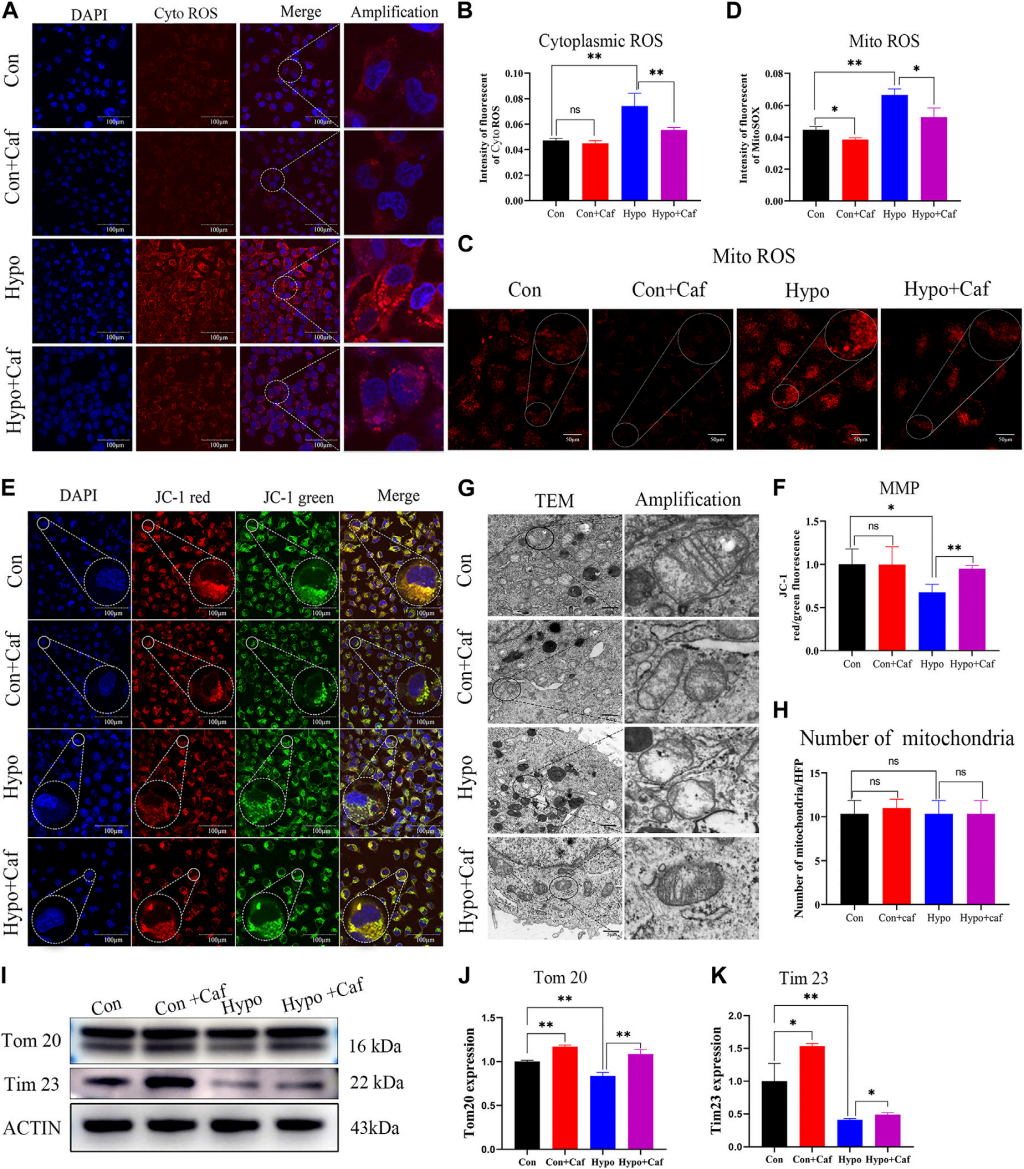
Caffeine reduces mitochondrial damage triggered by hypoxia and maintain mitochondrial morphology. **(A, B)** Confocal microscopy images of cytoplasmic ROS production (scale bars, 100 μm). The cytoplasmic ROS was measured using a fluorogenic probe (red), and the nucleus was marked by DAPI staining (blue). The intensity of fluorescent ROS was calculated by ImageJ software. Hypoxia caused a substantial increase in ROS production, but caffeine suppressed this ROS accumulation. **(C, D)** Confocal microscopy images of mitochondrial ROS production (scale bars, 50 μm). The mitochondrial ROS was detected by MitoSOX™ Red mitochondrial superoxide indicator. Hypoxia caused a significant increase in mitochondrial ROS production, but caffeine suppressed this ROS accumulation. **(E, F)** Confocal microscopy images of MMP (scale bars, 100 μm). MMP was measured using JC-1 reagent. The intensity of fluorescent of MMP. MMP is indicated by a ratio of the red/green fluorescence intensity. MMP decreased in hypoxia, while most mitochondria still have normal MMP in the caffeine group. **(G)** Representative transmission electron microscopy images of mitochondria at magnification 12000. Scale bars represent 5 μm. Mitochondria in the Hypo group showed more apparent cristae rarefication and swelling, wherein mitochondrial ultrastructure was severely disorganized. Caffeine can recover the conformation of mitochondria into normal in hypoxia. **(H)** The number of mitochondria per high-power field in each group. The number of mitochondria per field was counted and showed no difference in each group. **(I–K)** Representative Western blot images and summarized data of Tom20 and Tim23. ACTB was used as an internal reference. *p < 0.05, **p < 0.01.

To determine whether caffeine repaired the mitochondria damage caused by hypoxia, we detected the production of ROS and the changes in MMP. Previous studies have shown that damaged mitochondria will increase ROS production, decrease MMP and injure ATP synthesis (Labuschagne et al., 2019). We found that hypoxia significantly increased both the cytoplasmic ROS production and mitochondrial ROS production, but caffeine reduced the cytoplasmic and mitochondrial ROS accumulation (Figures 4A–D). MMP is a crucial parameter for evaluating mitochondrial function. MMP depolarization indicates cells become less healthy (Sakamuru et al., 2016). Oxidative stress damage caused the depolarization of MMP and further aggravated mitochondrial dysfunction. We found MMP got significantly depolarized in hypoxia, while caffeine maintained the normal MMP (Figures 4E,F). These results suggested that mitochondrial damage and oxidative stress triggered by hypoxia could be relieved by caffeine.

### 3.4 Caffeine reduces oxidative stress and stabilizes mitochondrial morphology

We found abnormally shaped mitochondria in the Hypo group compared to the typical conformation seen in the Con group in the transmission electron microscopy analysis (Figure 4G). Notably, mitochondria in the Hypo group showed more apparent cristae rarefication and swelling, wherein mitochondrial ultrastructure was severely disorganized. Caffeine normalized the damaged mitochondrial conformation in hypoxia (Figure 4G). While the number of mitochondria per high-power field showed no difference in each group (Figure 4H). We also found that Hypoxia increased the degradation of mitochondrial membrane proteins Tom20 and Tim23 (Figures 4I–K), reflecting the mitochondrial damage (Narendra et al., 2008). But caffeine attenuated the degradation (Figures 4I–K). These results indicated that caffeine protected the morphology and maintained the health of mitochondria.

### 3.5 Mechanism of the mitochondrial protective effects of caffeine

**FIGURE 5:**
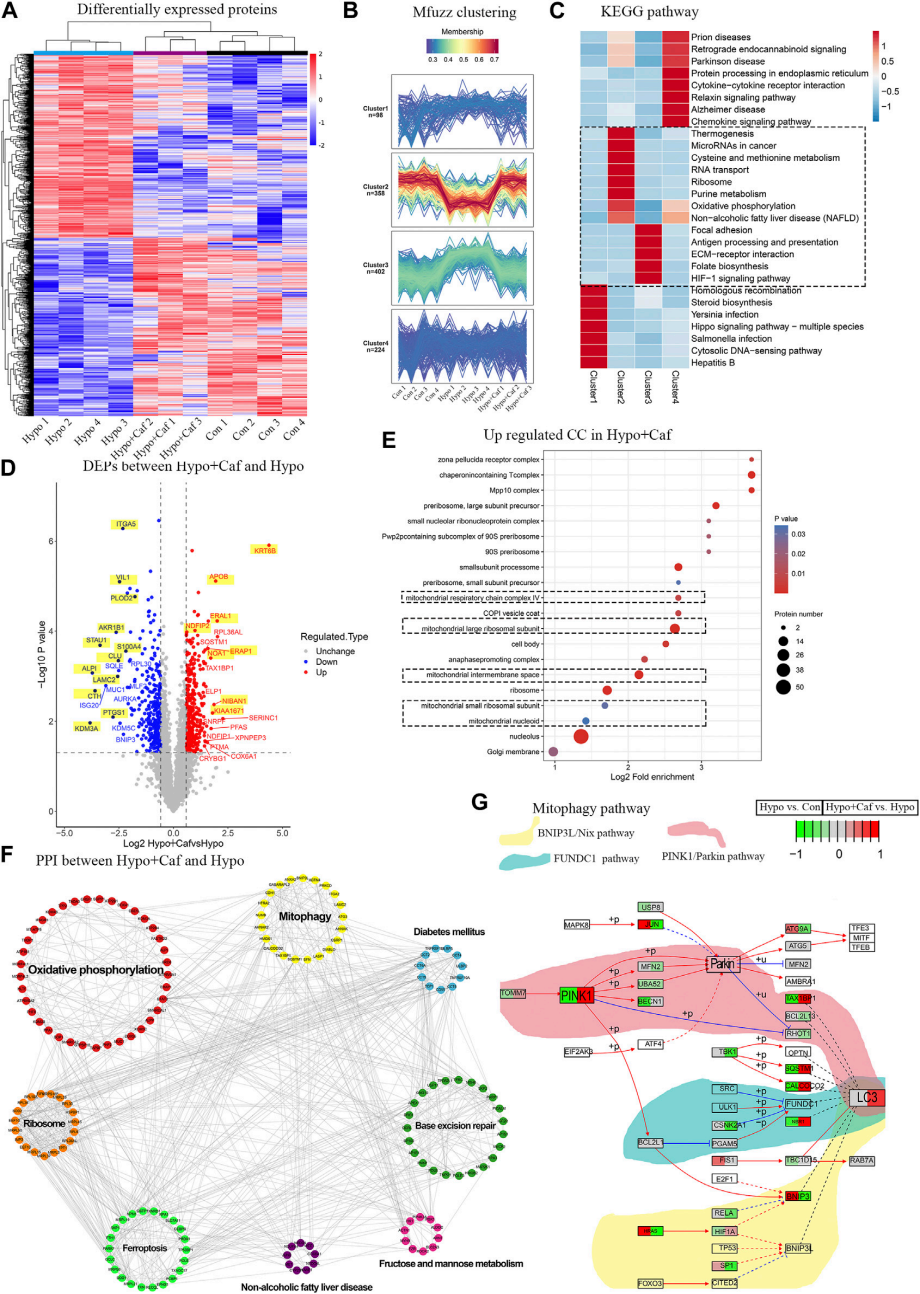
Mechanism of the mitochondrial protective effect of caffeine. **(A)** Heatmap of the quantitative proteomics. The heatmap with hierarchical clustering demonstrated Hypo + Caf group was closed to the Control group instead of the Hypo group, and caffeine could essentially recover the expression changes of the DEPs induced by hypoxia, indicating caffeine could relieve the damage caused by hypoxia. One sample in the Hypo + Caf group was removed due to the quality control of the proteomic data. **(B)** Mfuzz clustering analysis of DEPs. Mfuzz clustering analysis revealed four groups of proteins consisting of 10898, 541358, 428 402, and 431 224 quantified proteins, respectively. **(C)** The enrichment KEGG pathways of four clusters. Proteins in cluster 2 were downregulated in the Hypo group but increased in the Hypo + Caf group. In this cluster, oxidative phosphorylation was highly enriched. Proteins in cluster 3 were upregulated in the Hypo group but decreased in the Hypo + Caf group. The HIF-1a signaling pathway, ECM receptor interaction, thiamine metabolism, folate biosynthesis, protein digestion, and absorption were enriched in cluster 3. **(D)** Volcano map of DEPs between Hypo group and Hypo + Caf group. For these top 20 DEPS, we found that 60% of upregulated DEPs by hypoxia were decreased by caffeine, and 40% of downregulated DEPs by hypoxia were increased by caffeine. Co-regulated DEPs labeled with a yellow background color. **(E)** The cellular compound of GO enrichment analysis of the upregulated DEPs between Hypo + Caf group and Hypo group. The cellular component of the upregulated DEPs between the Hypo + Caf group and Hypo group were significantly enriched in mitochondria (dashed box). **(F)** Protein interaction network of DEPs between Hypo + Caf group and Hypo group. Eight significant modules of DEPs were visualized *via* the Cytoscape MCODE app, and the mitochondria-associated oxidative phosphorylation and mitophagy were revealed. **(G)** The specific expression level of DEPs in the mitophagy pathway. Most proteins in the mitophagy pathway were downregulated in the Hypo group (the left side of the box), but caffeine could increase the expression of these proteins in hypoxia (the right side of the box).

To investigate the mechanism of caffeine protective effects on mitochondria, we further implemented the label-free quantitative proteomics analysis in AT1 cells. We detected 104729 peptides and 7571 proteins (Supplementary Figure S3A). PCA showed that the three groups were separated, and the Hypo + Caf group was closer to the Con group. (Supplementary Figure S3B). 821 DEPs between the Hypo and Con groups and 687 DEPs between the Hypo + Caf and Hypo groups were detected (Supplementary Figure S3C; Supplementary Table S1). The volcano map of DEPs was shown, and the top 20 DEPs were labeled (Figure 5D; Supplementary Figure S3D). For these top 20 DEPs, we found that caffeine could markedly reverse the gene regulation induced by hypoxia. 60% of upregulated DEPs in hypoxia were decreased by caffeine, and 40% of downregulated DEPs in hypoxia were increased by caffeine (Figure 5D; Supplementary Figure S3D). The heatmap with hierarchical clustering demonstrated that the Hypo + Caf group was close to the Con group instead of the Hypo group (Figure 5A). Mfuzz clustering analysis revealed four clusters consisting of 98, 358, 402, and 224 quantified proteins, respectively (Figure 5B). Proteins in cluster 2 were downregulated in hypoxia but increased in Hypo + Caf group. In this cluster, oxidative phosphorylation was highly enriched (Figure 5C). Proteins in cluster 3 were upregulated in hypoxia but decreased in the Hypo + Caf group. The HIF-1a signaling pathway, ECM receptor interaction, and folate biosynthesis were enriched in cluster 3 (Figure 5C). From these clustering results, we found several pathways associated with energy metabolism and mitochondrial functions. The GO analysis of the upregulated DEPs between the Hypo + Caf and Hypo groups was significantly enriched in the ribosome (Supplementary Figure S3F), mitochondria (Figure 5E), and constituent of ribosome (Supplementary Figure S3G). Eight significant modules of DEPs were visualized *via* the Cytoscape MCODE app (Figure 5F), and the mitochondria-associated oxidative phosphorylation and mitophagy were marked. The suppressive oxidative phosphorylation manifests mitochondrial impairment (Brunetti et al., 2021), and mitophagy is one of the mechanisms to remove damaged mitochondria (Shan et al., 2019). We found that the upregulated KEGG pathway enriched the mitophagy pathway between the Hypo + Caf and Hypo groups (Supplementary Figure S3G). Most proteins in the mitophagy pathway were upregulated by caffeine could increase the expression of these proteins in hypoxia (Figure 5G). Therefore, we hypothesized that caffeine reduced mitochondrial damage in hypoxia by regulating mitophagy.

### 3.6 Caffeine mediated the mitochondrial quality control process

**FIGURE 6:**
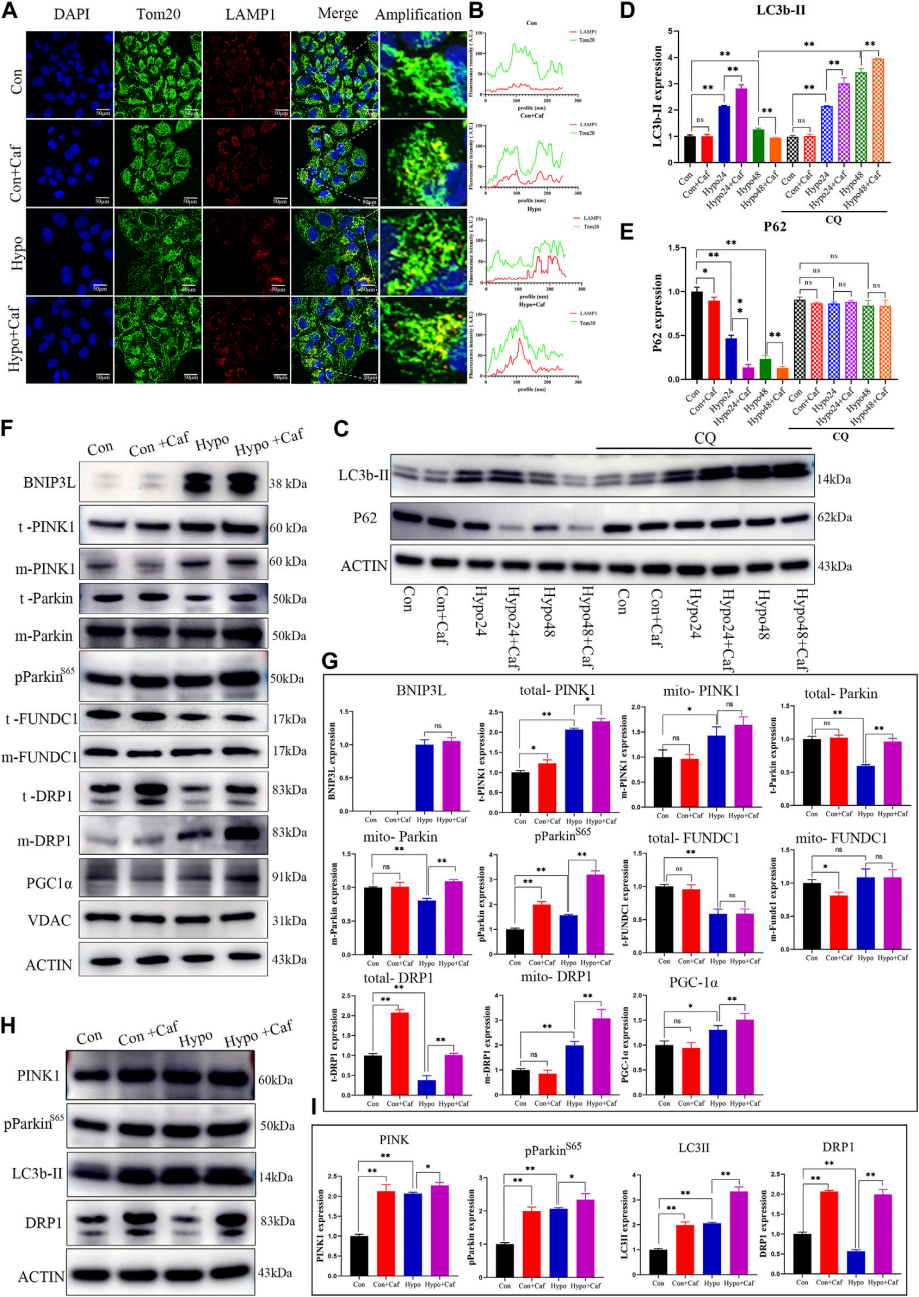
Caffeine mediated mitochondrial quality control process. **(A, B) (A)** The fluorescence images showed the colocalization of mitochondria (green fluorescence) and lysosomes (red fluorescence). The merged fluorescence (yellow) reflected mitophagy, and caffeine increased mitophagy in hypoxia. (Scale bars, 100 μm). **(B)** Quantification of colocalization. **(C–E)** Representative Western blot images and summarized data of LC3b-II and P62 following hypoxic treatment for 24h/48 h in the absence or presence of chloroquine (50 μM). LC3-II was significantly increased at the early stage of hypoxia treatment (24 h) and then gradually decreased (48 h), while caffeine further amplified the LC3-II changes. P62 protein expression level was downregulated in hypoxia, and caffeine enlarged the degree of decrease. CQ blocked the role of caffeine in the enhanced autophagic flux. **(F, G)** Representative Western blot images and summarized data of BNIP3L, t- PINK, m- PINK, t- Parkin, m- Parkin, pParkin (S65), t- FUNDC1, m- FUNDC1, t- DRP1, m- DRP1and PGC1α in AT1 cells. VDAC was the internal reference of mitochondrial membrane proteins, and ACTB was the internal reference of total proteins. **(H, I)** Representative Western blot images and summarized data of PINK, pParkin (S65), LC3II, and DRP1 proteins of lungs. ACTB was used as an internal reference.

As damaged mitochondria need to be cleared, we next detected whether caffeine contributes to the clearing of low-quality mitochondria, which are known to be removed through multiple ways, such as cell apoptosis, autophagy, mitophagy and mitocytosis (Pickles et al., 2018; An et al., 2021; Jiao et al., 2021; Song et al., 2021). Mitophagy is a particular form of autophagy to clear the damaged mitochondria and maintains cell homeostasis (Li et al., 2018). We examined the immunofluorescence colocalization analysis of mitochondria and lysosomes. Mitochondria displayed a regular uniform network in the control group (Figure 6A, green fluorescence). While hypoxia resulted in mitochondria fragmentation and increased colocalization of mitochondria and lysosomes (red fluorescence), which reflected increased mitophagy (yellow fluorescence) (Figure 6A). The yellow fluorescence was intensive in the Hypo + Caf group (Figure 6A). The quantification of colocalization showed that caffeine enhanced the colocalization of Tom20 and LAMP1 (Figure 6B), which suggested that caffeine improved mitophagy in hypoxia. LC3 is a mammalian autophagosome with two isoforms, LC3-I and LC3-II. The conversion of the cytosolic-associated protein LC3-I to the membrane-bound LC3-II form is an important indicator of autophagosome activation. Therefore, the detection of LC3-II can be used to evaluate autophagosome formation (Bjorkoy et al., 2009). Western blot analysis showed that the expression level of LC3-II was significantly increased at the early stage of hypoxia treatment (24 h) and then gradually decreased (48 h). Caffeine treatment amplified the changes in LC3-II expression levels under hypoxic conditions. (Figures 6C,D). In addition, P62 was integrated into the formed autophagosome and degraded in autolysosomes, which was another common marker to study autophagic flux (Bjorkoy et al., 2009). The protein expression of p62 was decreased in hypoxia, suggesting enhanced autophagy. Compared with the Hypo group, caffeine further aggravated P62 degradation in hypoxia (Figure 6C; Figure 6E). Chloroquine (CQ) was an inhibitor of the fusion of autophagosomes and lysosomes, was used to further investigate the role of caffeine in the induction of mitophagy. We found that the enhanced autophagic flux induced by caffeine was inhibited by CQ (Figure 6C), which suggested that caffeine increased mitophagy by promoting autophagolysosome degradation.

Given the three classic pathways in mitophagy, we detected the expression of essential proteins in these pathways. BCL2/adenovirus E1B interacting protein 3 like (BNIP3L/Nix), a mitochondrial protein, plays a critical role in mitophagy (Fu et al., 2020). BNIP3L-mediated mitophagy is activated by HIF-1a in hypoxia (Lin Q. et al., 2021). BNIP3L protein showed low-level expression in normoxia and significantly increased in hypoxia. In comparison, we found that caffeine did not affect the expression of BNIP3L (Figures 6F,G). The second pathway is the PINK1/Parkin-dependent mitophagy pathway, which is the primary mechanism of mitochondrial membrane depolarization-induced mitophagy (Poole and Macleod, 2021). We found that hypoxia increased the expression of the total PINK1(t- PINK) and mitochondrial PINK1(m- PINK), and caffeine enlarged the degree of increase in hypoxia. Parkin is auto-inhibited and requires activation by PINK1, which phosphorylates Ser65 in the ubiquitin domain (Tan et al., 2022). The mitochondrial Parkin (m- Parkin) and phosphorylated Parkin (pParkin) were increased in hypoxia, and caffeine magnified this increased degree (Figures 6F,G), suggesting caffeine enhanced the PINK1/Parkin pathway in hypoxia. PARKIN phosphorylation is a signal to activate its E3 ligase activity, which leads to the ubiquitination of the VDAC, TOM20, COXII, MFNs, and multiple other mitochondrial membrane proteins to label the damaged mitochondria for mitophagy (Narendra et al., 2008). We found that damaged mitochondria, marked by parkin puncta (Saha et al., 2022), showed degradation of COXII protein under hypoxia and caffeine treatment (Supplementary Figure S4). The total FUNDC1 (t- FUNDC1) expression decreased in hypoxia, but caffeine did not affect its expression in hypoxia. The expression of mitochondrial FUNDC1 (m- FUNDC1) was slightly reduced in normoxia by caffeine but showed no difference in hypoxia (Figures 6F,G). Most changes in protein expression levels were consistent with quantitative proteomics results and proved the stability of the results (Figure 5G). These findings suggested that mitophagy was increased in hypoxia, which was further amplified by caffeine *via* the PINK1/Parkin pathway.

Excessive mitophagy decreased the number of mitochondria and impaired ATP production. But the number of mitochondria did not lessen in the Hypo + Caf group (Figures 4I–K). This indicated that there must be other mechanisms to regulate the number of mitochondria by caffeine. Previous studies reveal that fission underlies both proliferation and degradation of mitochondria (Kleele et al., 2021). Thus, we hypothesized whether caffeine also regulated mitochondrial fission in hypoxia to maintain the mitochondria quantity. To verify this conjecture, we detected the expression of dynamin-related protein 1 (DRP1), which is the signature protein of mitochondrial fission. The expression of the total DRP1 (t- DRP1) was decreased in hypoxia, while caffeine could significantly increase its expression. The expression of mitochondrial DRP1 (m- DRP1) was increased under hypoxic conditions, which was amplified by caffeine (Figures 6F,G). Peroxisome proliferator-activated receptor-γ coactivator-1-α (PGC-1α) is a master indicator of mitochondrial biogenesis (Li et al., 2017). The expression of PGC1α was increased in hypoxia, which was amplified by caffeine (Figures 6F,G). We next explored the mitophagy and mitochondrial fission markers in mice models. Consistently with the result in AT1 cells, the expression of PINK, pParkin (S65), LC3II, and DRP1 of lungs was also upregulated by caffeine, especially in hypoxia (Figures 6H,I). These results indicated that caffeine mediated mitochondrial quality control by activating mitophagy, mitochondrial biogenesis, and mitochondrial fission to mitigate the damage caused by hypoxia.

## 4 Discussion

High altitude pulmonary edema (HAPE) as one of the severe sub-type of high altitude illness is considered a life-threatening disease (Swenson and Bartsch, 2012; Wang Y. et al., 2022), but the effectiveness of current therapies was unsatisfactory (Luks et al., 2019; Luks and Hackett, 2022). Caffeine is a small molecule compound with biological activity, which can be used to treat respiratory distress syndrome (Ines et al., 2021; Elmowafi et al., 2022). HAPE has similar pathological changes to respiratory distress syndrome, but there is no report on whether caffeine plays a role in alleviating HAPE. In our study, we found that a low dose of caffeine could relieve HAPE through mediating the mitochondrial quality control to reduce oxidative stress in AT1 cells.

Through a series of experiments and bioinformatics analysis, we demonstrated that caffeine alleviates HAPE might by regulating the mitochondrial oxidative phosphorylation (OXPHOS) pathway. Previous studies show that caffeine could enhance OXPHOS to increase mitochondrial bioenergetics, promoting the necessary energy supply for brain recovery (Goncalves et al., 2020). We found that caffeine neutralized the ATP reduction and significantly increased the glycolytic capacity of AT1 cells in hypoxia. Mammalian cells normally require molecular oxygen to produce ATP as an energy source through the OXPHOS of mitochondria. However, under hypoxic conditions, the energy source is mainly provided by glycolysis (Wang D. et al., 2022). Tibetan chickens are well-adapted to hypoxia, showing decreased oxygen consumption rates and increased glycolysis to enable adaptation to hypoxia compared with dwarf-laying chickens (Tang et al., 2021). We found that caffeine significantly increased ATP production, especially GlycoATP production, under hypoxic conditions. The expressions of rate-limiting enzymes in glycolysis, such as PFKP and PKM1/2, were upregulated by caffeine. These results revealed that caffeine maintained an adequate energy supply by promoting glycolysis under hypoxic conditions in AT1 cells.

The accumulation of ROS caused by hypoxia leads to oxidative stress damage, disruption of mitochondrial structure and mitochondrial permeability transition (Bugger and Pfeil, 2020; Chowdhury et al., 2020; Sreedhar et al., 2020). We found that caffeine reduced the production of cytoplasmic ROS and mitochondrial ROS induced by hypoxia. MMP plays an important role in maintaining mitochondrial stability, and MMP depolarization induced by oxidative stress can induce cell apoptosis (Yang et al., 2019; Lin B. et al., 2021). We found decreased MMP and swelled mitochondria in hypoxia, but caffeine suppressed the MMP depolarization and recovered the typical morphological characteristics of mitochondria.

Caffeine alleviated hypoxic damage in HAPE by regulating mitochondrial quality control. To maintain the quality of mitochondria, cells must remove the damaged mitochondria in multiple ways, such as cell apoptosis, autophagy, mitophagy, and mitocytosis (Gao et al., 2021; Hyttinen et al., 2021; Jiao et al., 2021). What is the mechanism by which caffeine maintains the mitochondrial function under hypoxia? The label-free quantitative proteomics of AT1 cells indicated that caffeine promoted mitophagy. Mitophagy is a particular form of autophagy that clears the damaged mitochondria to maintain cell homeostasis (Li et al., 2018; Onishi et al., 2021). We explored the expression of proteins in three classic mitophagy pathways and found that caffeine enhanced the PINK1/Parkin-dependent mitophagy. But excessive mitophagy will cause insufficient numbers of mitochondria and decreased cellular resistance to injury due to energy shortage (Wu et al., 2020). We found no lessen in the number of mitochondria in the hypoxic and caffeine-treated groups. Hence, we detected the expression of signature proteins of mitochondrial fission (DRP1) and mitochondrial biogenesis (PGC-1α) to explore the effect of caffeine on mitochondrial fission and biogenesis. Indeed, caffeine upregulated the expression of DRP1and PGC1α, indicating the upregulation of mitochondrial fission and biogenesis. Collectively, we found that caffeine maintained mitochondrial quality control by regulating the PINK1/Parkin-dependent mitophagy, mitochondrial fission, and biogenesis.

## 5 Conclusion

In conclusion, we found that low-dose of caffeine could relieve pulmonary edema in hypobaric hypoxia *in vivo*. Caffeine attenuated hypoxia-induced mitochondrial damage by reducing oxidative stress and restoring mitochondrial morphology *in vitro*. Caffeine maintained the mitochondria quality control by enhancing the PINK1/Parkin-dependent mitophagy, promoting mitochondrial fission and biogenesis under hypoxic conditions. Furthermore, our results suggest that caffeine alleviated HAPE *via* regulating mitochondrial dynamics in AT1 cells, but further research is needed to determine whether a low dose of caffeine can increase mitochondrial turnover in other types of alveoli cells.

## Data Availability

The raw proteomic data analyzed in this study are available at iProX with the corresponding dataset identifier PXD034002 (https://www.iprox.cn/page/project.html?id=IPX0004468000).

## Abbreviations

HAPE, High-altitude pulmonary edema; AHAI, Acute high altitude illness; HIF-1α, hypoxia-inducible factor-1α; VEGF, vascular endothelial growth factor; ROS, reactive oxygen species; RDS, respiratory distress syndrome; UPLC, ultra-performance liquid chromatography; LFQ, label-free quantitation; DEPs, differentially expressed proteins; GO, Gene Ontology; KEGG, Kyoto Encyclopedia of Genes and Genomes; AT1, Type Ⅰ alveolar epithelial; FBS, Fetal bovine serum; OCR, oxygen consumption rate; ECAR, extracellular acidification rate; 2-DG, 2-deoxy-D-glucose; MMP, mitochondrial membrane potential; PCA, Principal component analysis; DRP1, dynamin related protein 1; PGC-1α, peroxisome proliferator-activated receptor-γ coactivator-1-α.
