# Supplementary material for: Low-dose of caffeine alleviates high altitude pulmonary edema via regulating mitochondrial quality control process in AT1 cells
Source: Front Pharmacol. 2023 Apr 4;14:1155414. doi: 10.3389/fphar.2023.1155414 (PMC10110878; doi:10.3389/fphar.2023.1155414)
Supplement: Supplementary file 1 [file DataSheet1.DOCX]

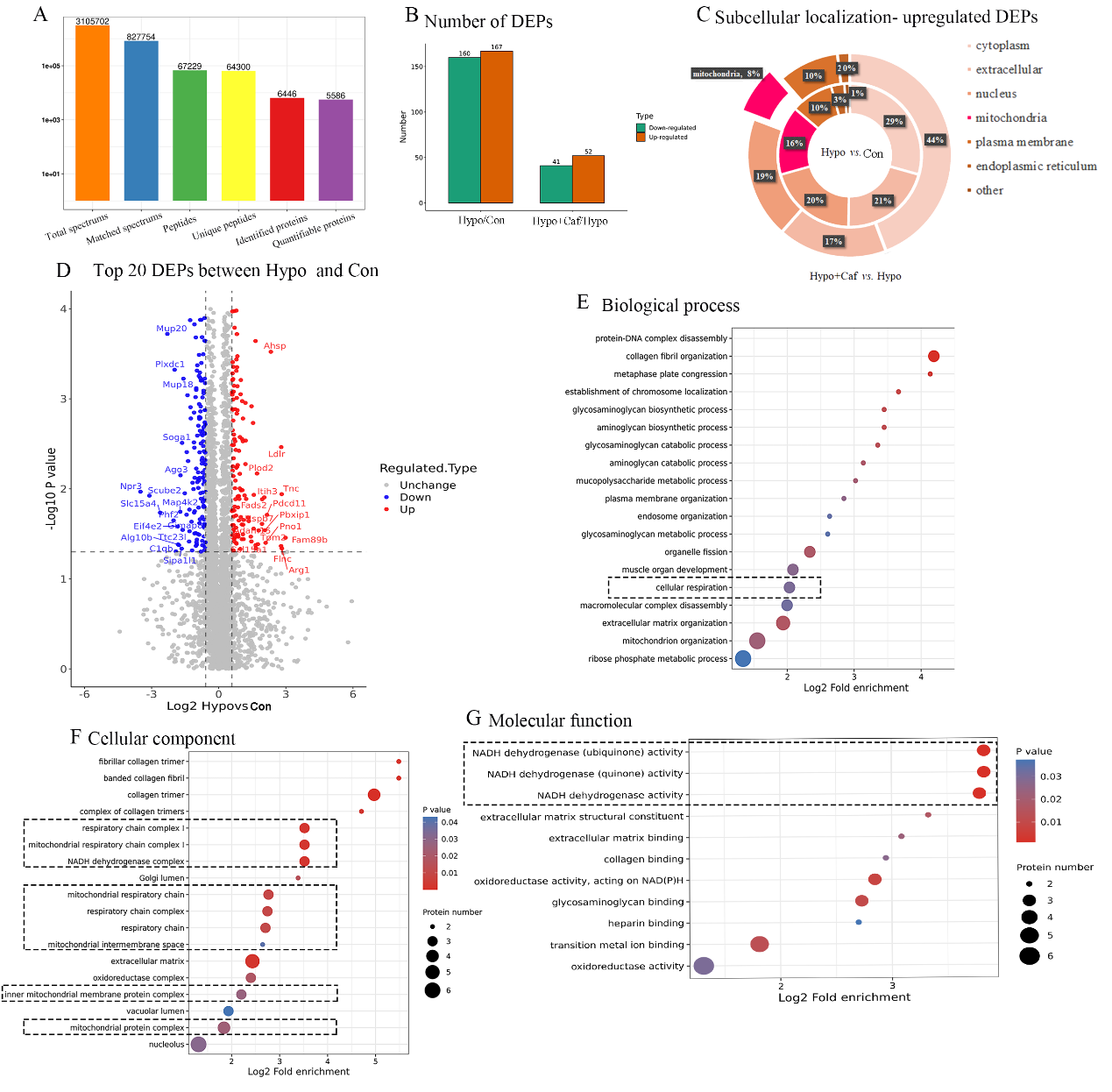


Supplementary Figure S1. Quantitative proteomics of mouse with HAPE.

(A) An overview of protein identification. We detected 6,446 identified proteins and 64,300 unique peptides from 3,105,702 MS spectra.

(B) The number of DEPs. We found 327 DEPs between the Hypo and Con groups and 93 DEPs between the Hypo+ Caf and Hypo groups.

(C) Subcellular localization of upregulated DEPs. The internal ring refers to the subcellular localization ratio of DEPS between the Hypo and Control groups. The external ring refers to the subcellular localization ratio of DEPS between the Hypo+ Caf and Hypo groups. 16% DEPs were upregulated in the Hypo group compared with the Con group in mitochondria (pink sector).

(D) Volcano map of DEPs between the Con and Hypo groups. Names of the top 20 DEPs were labeled.

(E-G) Gene Ontology (GO) enrichment analysis of the downregulated DEPs between the Hypo and Hypo+ Caf groups. These DEPs were significantly enriched in cellular respiration (BP), mitochondrial respiratory complexes (CC), and NADH dehydrogenase oxidative phosphorylation (MF).


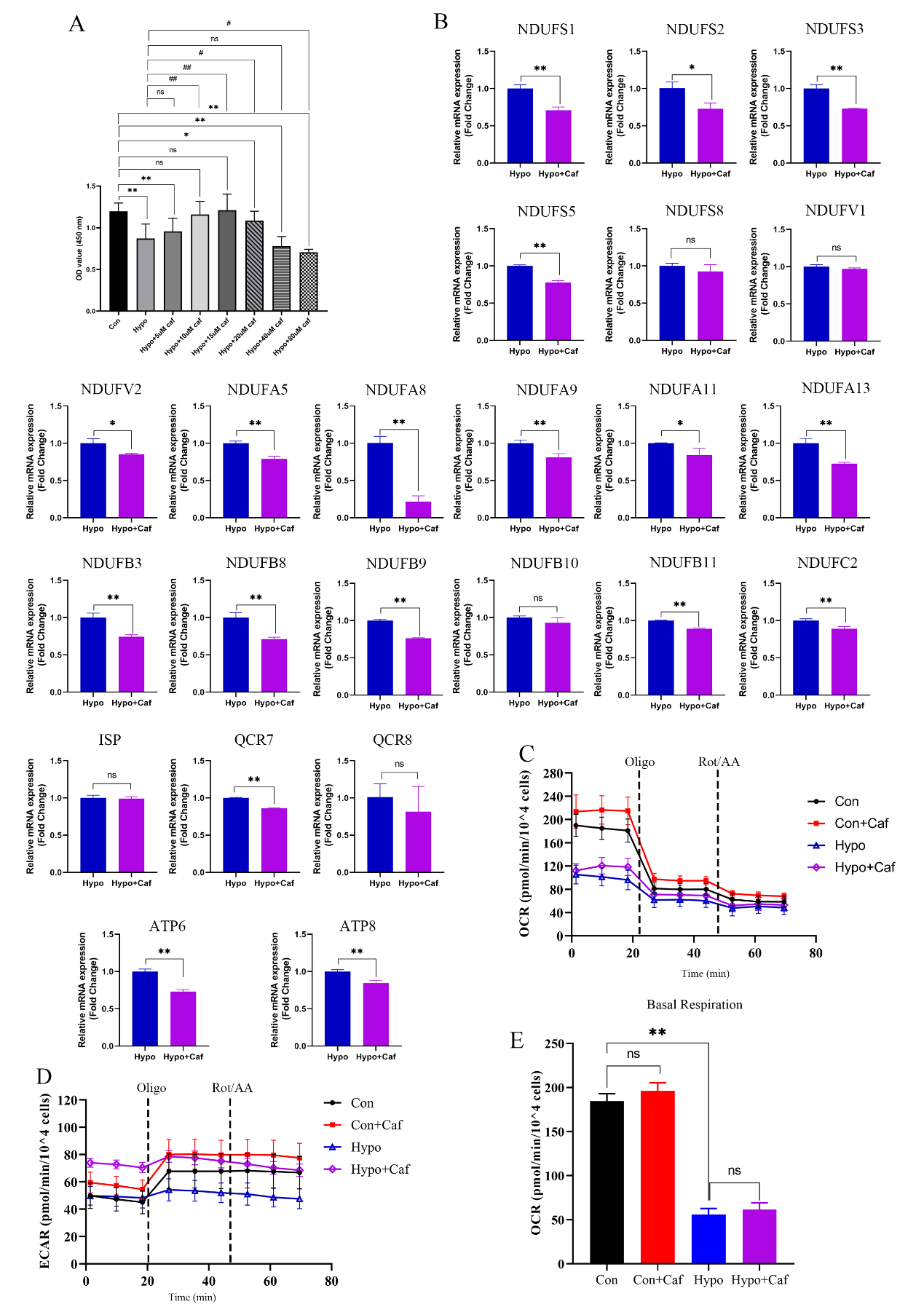


Supplementary Figure S2. Effects of caffeine on mitochondrial function.

(A) The CCK8 assay detected the cell viability effect of caffeine. Hypoxia decreased the cell viability, and caffeine increased the cell viability with a dose-dependent. 15μM caffeine was the optimum concentration. **P*<0.05, ***P*<0.01 compared with Con group, ^#^*P*<0.05, ^##^*P*<0.01 compared with Hypo group.

(B) The relative mRNA expression of the genes in the mitochondrial oxidative phosphorylation pathway. Caffeine decreased the expression of these genes in hypoxia. **P*<0.05, ***P*<0.01.

(C) The oxygen consumption rate of mitochondria. By measuring the oxygen consumption rate (OCR), we calculated the amount of ATP produced by mitochondrial oxidative phosphorylation (mitoATP).

(D) The rate of extracellular acidification. By measuring the extracellular acidification rate (ECAR), we calculated the amount of ATP produced by glycolysis (glycoATP).

(E) Basal respiration of the Mito Stress Test was calculated. Hypoxia decreased basal respiration, but caffeine did not affect it. ***P*<0.01.


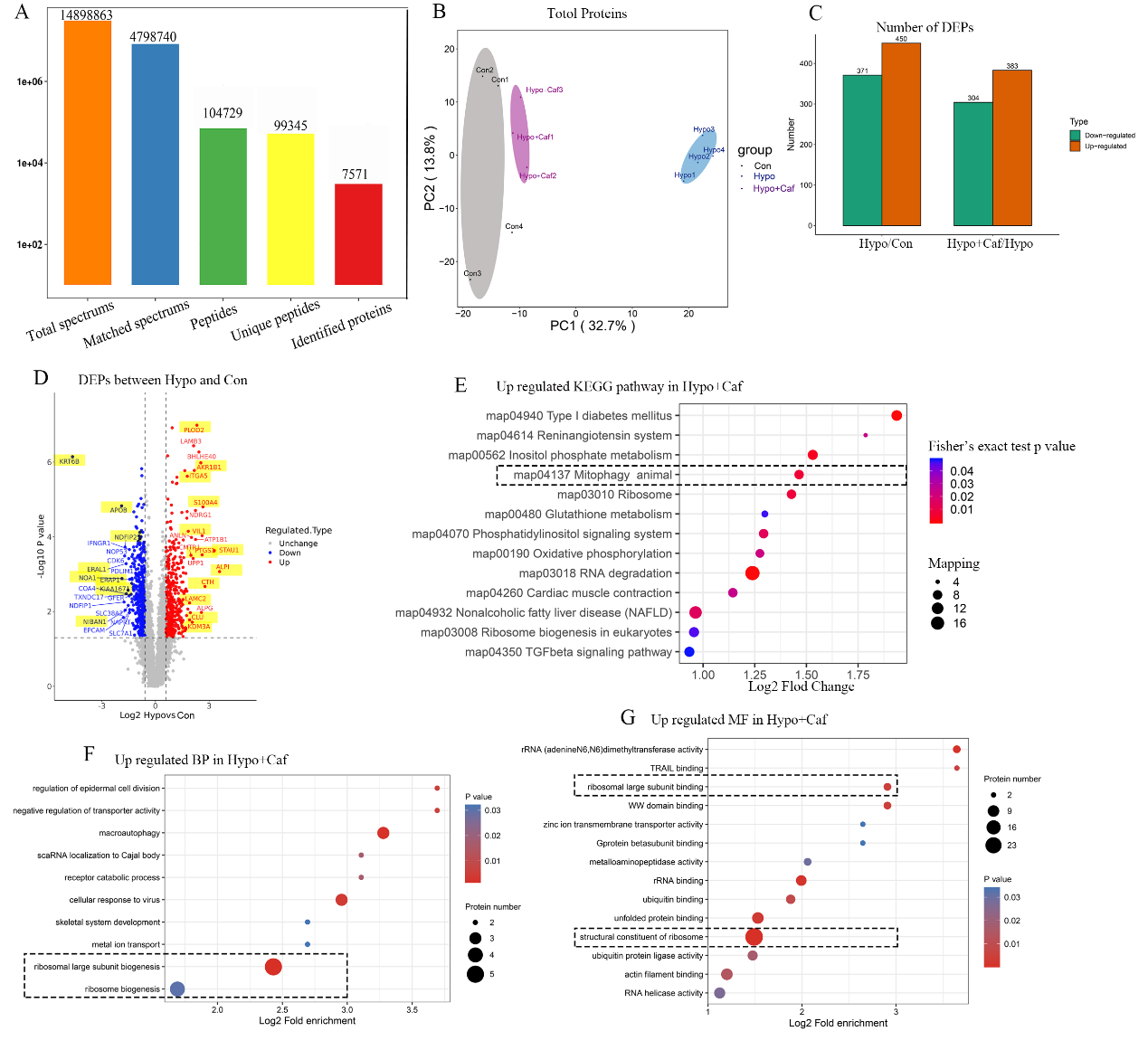


Supplementary Figure S3. Quantitative proteomics of AT1 cells in hypoxia.

(A) An overview of protein identification. We detected 7,571 identified proteins and 104,729 unique peptides from 14,898,863 MS spectra.

(B) PCA of 11 samples based on the expression of all DEPs. PCA showed these three groups separated from each other, and the Hypo+ Caf group was close to the Con group.

(C) The number of DEPs. There are 821 DEPs between the Hypo and Con groups, and 687 DEPs between the Hypo+ Caf and Hypo groups.

(D) Volcano map of DEPs between the Con and Hypo groups. For these top 20 DEPS, we found that 60% of upregulated DEPs caused by hypoxia were decreased by caffeine, and 40% of downregulated DEPs caused by hypoxia were increased by caffeine. Co-regulated DEPs were labeled with a yellow background color.

(E) The enrichment KEGG pathway of the upregulated DEPs between the Hypo and Hypo+ Caf groups. The upregulated KEGG pathways of DEPs were enriched in the mitophagy pathway.

(F-G) The biological process and molecular function of GO enrichment analysis of the upregulated DEPs between the Hypo and Hypo+ Caf groups. The GO analysis of the upregulated DEPs between the Hypo+ Caf and Hypo groups was significantly enriched in the biogenesis and constituent of ribosome.


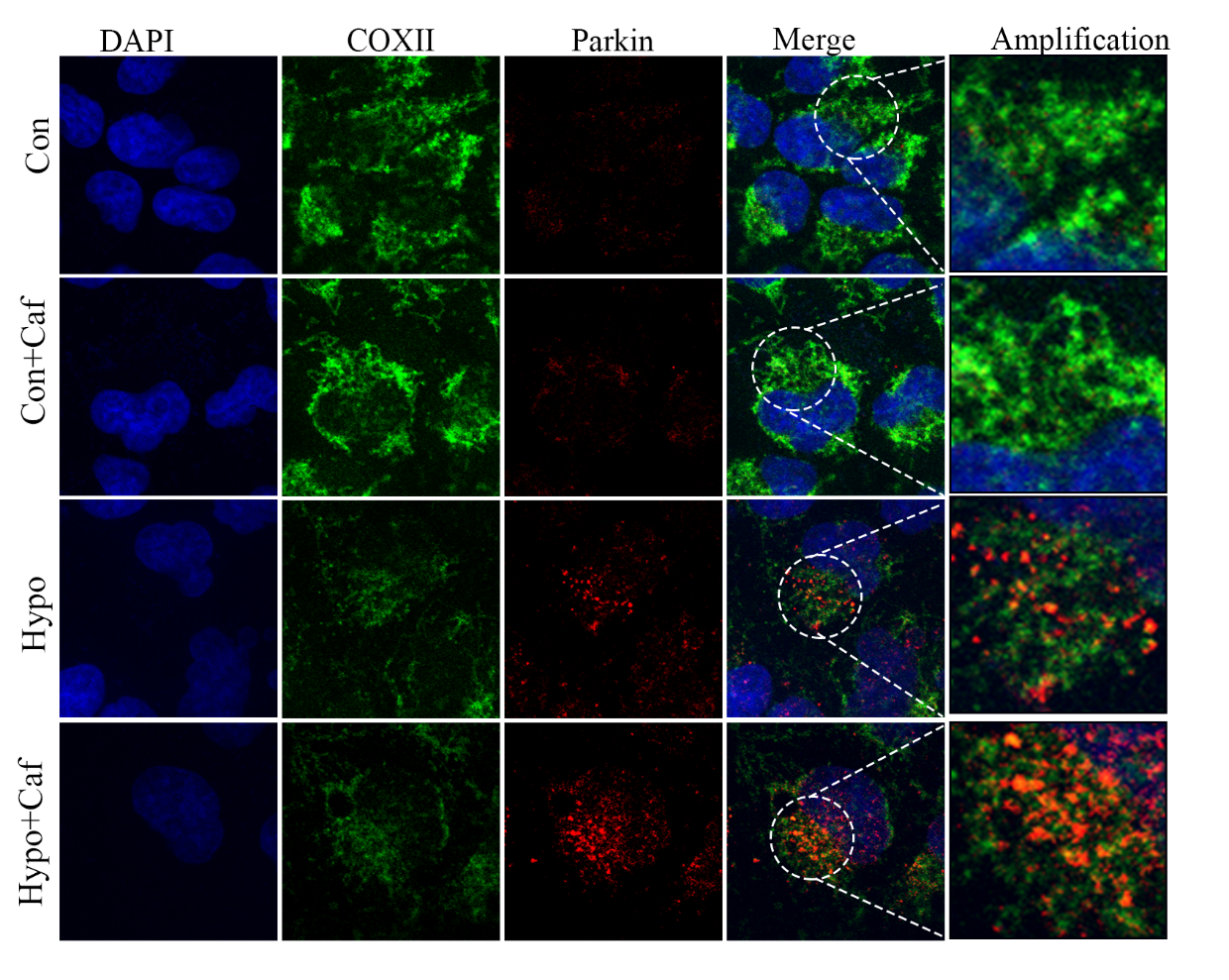


Supplementary Figure S4. The degradation of mitochondrial COXII protein in damaged mitochondria in hypoxia. The fluorescence images showed the colocalization of damaged mitochondria (red fluorescence) and COXII (green fluorescence). (Scale bars, 100 μm). The level of COXII was decreased within damaged mitochondria

| group | Gene name |
| --- | --- |
| Hypo/Con upregulated DEPs (167) | Tnni2; Myom3; Fam89b; Arg1; Tnc; Ldlr; Flnc; Ahsp; Pdcd11; Pno1; Pbxip1; Itih3; Hmgcs1; Fads2; Hspb7; Adam15; Lss; Tpm2; Plod2; Col15a1; Sult1a1; Coq8b; Aldoc; Apobec3; Rrp12; Col3a1; Rrm1; Tubb3; Tep1; Cirbp; Sorcs2; Rbm3; Filip1l; Tfrc; Fbln2; Tbce; Mcm4; Fkbp10; Ckmt1; Tubb6; Samd9l; Fbxo3; Lox; Fndc3b; Crtap; Gpx2; F3; Kpna2; Aldoa; Wdr43; Igfbp2; Itga7; Ubr3; Mvk; Fn1; Psat1; Lrp2; Mrps25; Hk2; Col12a1; Tmlhe; Mcm5; Gas7; Hmox1; Mbl2; Trex1; Ugdh; Dysf; Apoh; Parp3; Hmgcs2; Dnajc16; Idi1; Dcaf1; Plin2; Tpt1; Hnrnpdl; Nsdhl; Sema7a; Dtymk; Tbc1d1; Tgfbi; Eny2; Mrpl24; P4ha2; Itga5; Hrg; Podn; Tagln; Epha2; Rcn3; Hebp1; Ctps1; Ddx39a; Npm3; Mcm6; Fetub; Aldh18a1; Mat1a; Eif2s3y; Ipo4; Pcolce; Sec11c; Polr1c; Mcm7; Dnajc25; Sparc; Rplp1; Mrps10; Entpd5; Itih1; Mcm2; F12; Itih2; Nes; Mrc1; Serpina3m; Mcmbp; Glrx2; Cspg4; Col1a1; Myh11; Asns; Lgals3bp; Mvd; Mthfd1l; Mrpl49; Hmbs; Fam162a; Mrps28; Mgll; Plod1; Prkra; Hsd17b7; Mcm3; Timm23; Nt5c3a; Plod3; Impad1; Trim47; Nsun2; Eppk1; C4bpa; Pdlim7; Gfpt1; Dbn1; Mpst; Dab2; Acp2; Pde12; Jmjd6; Lgals1; Cpox; Pcm1; Fdps; Aars1; Erlec1; Uap1; Egflam; Rabac1; Gpld1; Kng1; Aldh1a2; Ikbip; Ptgr1; Gfm1; Clptm1 |
| Hypo/Con downregulated DEPs (160) | Npr3; Scube2; Hpgd; Slc15a4; Mup20; Iglc2; Lifr; Phf2; Plxdc1; C1qb; Eif4e2; Alg10b; Ttc23l; Kit; Map4k2; Ago3; Sipa1l1; Soga1; Gimap8; Mup18; Eng; Sema3c; Angptl2; Ociad2; Tek; Gzma; Frg1; Pten; Pacs2; Vnn1; Ntn1; Bmpr2; Rpl39; Paxbp1; Mturn; Apbb2; Ppp1r14c; Ushbp1; Brd3; Bst2; Vsnl1; Rpl26; Hmgb3; Nfia; Naalad2; Rpl24; Zbtb20; Frmd4a; C9; Znf512; Serpina1e; Ercc4; Zranb2; H2-Q8; C1sa; Pm20d1; Kiaa1324; Npnt; Spint2; Mcf2l; H3-5; Dr1; Ubqln4; Saa4; Plpp3; Gngt2; Dhrs3; Cfd; Oma1; Kank4; Hmbox1; Hfe; Ces1; Hp1bp3; Nostrin; Dgkz; H4c1; Emp2; Antxr2; Myzap; Clec3b; She; Ubxn7; Gucy1a1; Plvap; Bfar; Angpt2; Cdkn1b; Cyth3; Reck; Pltp; Ece1; Ado; Gbp4; Agap3; Pid1; Itm2b; Comtd1; Plekha7; Vangl1; Trim2; Syde1; P2rx7; Ces2e; Top1; C8a; Tmcc3; Slc29a1; Pum1; Mpp2; Prex2; Mxra8; Adgre5; Hsph1; Rasgrf2; Npr1; Ints14; Mndal; Fkbp4; Stxbp6; Lama3; Eif4e3; Map2k6; Tcn2; Tax1bp1; Thbd; Stxbp5; Psen1; Macroh2a1; Radil; Lamb3; Cggbp1; Aplp2; Snx33; Setd7; Fgf1; Smug1; Pde3a; Enpp5; Tmcc2; Luc7l; Arhgap25; Pld4; Brd4; Evl; Nr3c1; Sash1; Ap3s2; Plcg1; Stx6; Gucy1b1; Cdkn2aip; Tpk1; Faim; Sp100; Palm; Borcs7; Dop1b; Dnaja1; Gtf2b |
| Hypo+Caf/Hypo upregulated DEPs (52) | Mpo; Rpl35; Arl6; Ebna1bp2; Rpl27a; Spns2; Rpl27; Rpl24; Rpl26; Borcs8; Rpl15; Rpl32; Rpl37a; Slfn14; Rpl19; Rpl8; Saa4; Rpl28; Rpl13a; Rpl35a; Rps29; Edf1; Rpl23a; Rpl3; Rps24; Rpl36a; Arrb2; Hykk; Rps8; Rpl21; Timp3; Rps26; Rps25; Rps11; F10; Cpb2; Rpl17; Lsm14a; Arfgap3; Plekha1; Rpl23; Lifr; Rps14; Uhrf1bp1l; Rpl31; Lpin2; Apoa1; Cpn2; Ap3s1; Emp2; Gimap8; Hp1bp3 |
| Hypo+Caf/Hypo downregulated DEPs (40) | Ggh; Pno1; Polr2l; Col15a1; Cd109; Cd180; Tpm1; Septin1; Prg2; Acsl3; Acta2; Itga7; Dcn; Aoc3; Atp6ap1; Entpd5; Pdcd11; Sec14l1; Use1; Grwd1; Eny2; Npm3; Mrps28; Rplp1; Lum; Fam89b; Dpt; Dnm1; Timm8a1; Dohh; Ndufb8; Col5a1; Susd2; Smarcb1; Nudt19; Glrx2; Ndufa10; Dysf; Ndufs1; Chmp1a |

Supplementary Table 1. The DEPS of pulmonary tissues in each group.

Supplementary Table 2. The DEPS of AT1 cells in each group.

| group | Gene name |
| --- | --- |
| Hypo/Con upregulated DEPs (450) | ALPI; STAU1; CTH; S100A4; VIL1; PTGS1; ALPG; AKR1B1; BHLHE40; PLOD2; ATP1B1; NDRG1; ITGA5; LAMB3; UPP1; KDM3A; ANLN; CMTR1; CLU; LAMC2; HMGN3; NUSAP1; SAMHD1; ALPP; NCOA7; ARG2; MUC1; SKP2; ALKBH5; AGR2; LIG1; EBP; MLF2; CENPF; LAMB2; SLC2A3; STS; SEC24D; LIMCH1; CSRP2; ITPR1; PPIL2; ANK3; KDM4B; HBB; PNPLA6; SQLE; TSPO; SLC25A20; PLIN4; WEE1; P4HA2; UTRN; MSMO1; ZKSCAN8; CHD2; DDX60; RPS23; DNAJB6; B2M; COL6A1; MGST3; ALDOC; BNIP3; PHLDB2; TLE3; THEM6; MAGED2; RNASEH2C; DTX3L; TDRD7; DDX59; KANK2; DDX41; GTSE1; EDF1; PDK1; AKR1C3; EDIL3; NCAPG2; METTL3; DDA1; LNPK; TUBGCP2; ADARB1; AURKA; GTF2E2; CEBPB; KDM5C; GRB7; JAGN1; RPS6KA4; PRKRA; TPD52L1; PC; PIEZO1; SMARCAL1; NEDD1; GABPB1; SOD2; PAPOLA; ULBP2; ERO1A; HK1; C15orf40; CARD19; CEMIP2; RPA3; ISG20; P4HA1; CWF19L1; TSEN34; ARFGAP3; ANO6; KDM3B; MAPRE3; FERMT2; RO60; B3GAT3; RANBP3; PEDS1; S100A10; YTHDC2; HMGB2; TMBIM6; EPS8; SH3GLB1; PHF2; PCSK9; TPX2; ZRANB2; HLAE; AURKB; PBXIP1; COL7A1; PDIA5; DGCR8; MYH10; REPIN1; JUND; BZW1; LRATD2; KLF5; GSTM3; PRIM1; SUSD2; PDRG1; APOBEC3B; KIF2A; MIDEAS; PLSCR1; KIF20A; KIFC1; JUN; PHC2; ACSF2; MTCO1; SEMA3B; RPIA; RRAS; GGH; MBNL2; PET100; CTTNBP2NL; MELTF; CTSL; SMDT1; TNFAIP2; TMEM109; WNK1; TEAD1; BCKDHA; AGRN; THYN1; CLK1; SBNO1; TLN2; GTF2E1; HSPA14; HDGF; TXNRD2; SUN2; EIF4A2; CSNK1D; VPS13B; F3; ZBED1; GNG12; WRNIP1; SLC2A1; CAMK2G; FOSL2; PCIF1; CCNB1; MORF4L2; TUBGCP3; SEC24A; TOP2A; ZFC3H1; CDC42BPB; RPRD1A; TMEM263; FDX1; RAB5B; NFKB2; HSDL1; CCNA2; GABARAP; DLG5; MPG; RNASEH2B; NFYC; PRC1; IDH2; PICALM; ERI1; SH3KBP1; EPHX1; ARHGAP5; VPS53; MAP1S; LEO1; C4orf3; IL1RAP; COG3; RPL36; TMEM65; TNS4; RECQL; NFIA; NELFA; CRYBG2; KHDC4; HELZ2; USP11; MANF; PRRC1; PON2; IRF2BPL; DNAJB4; DUSP3; NUDCD1; CARMIL1; POLD3; RBM12; FAM50A; BRD8; MORF4L1; HLTF; POFUT2; CAMLG; ERG28; ANKZF1; LSS; MTOR; WIPF2; CMAS; BCKDK; UBE2I; OAS3; RNGTT; TACC3; UGGT2; DCAF1; ZMYND8; DPYSL2; HPCAL1; RBBP5; ECT2; TAP2; ADD1; ABHD12; CUL4B; DEGS1; STAU2; SAP18; KIF22; HTRA1; H110; DSCC1; OCRL; SPIN1; POLR2F; CDC42EP4; PIK3C2A; IWS1; MPC2; MET; PHF6; FAM234A; GPRC5A; BDH1; SPATS2; WASL; RPA1; HNRNPUL2; ATAD2; LLGL1; MCM3; PCBP1; GRB10; MSH2; FEN1; SYNE2; CCDC127; MORC2; MCM2; SUPT4H1; INCENP; HNRNPLL; ZC3HC1; ITGA2; TEX10; ATP5MK; SBDS; PML; RPTOR; CNNM3; SMAD3; DDX17; HMCES; HACD3; NFATC2IP; NUMB; TAP1; CCNT1; MCM6; DOCK5; PRIM2; ARHGAP18; PLEK2; CDK9; GAPDH; WDR61; FKBP3; SIPA1; MCM7; PIN4; ST3GAL4; SRPRA; APMAP; PTP4A2; EPB41L1; ACADSB; ERMP1; BCAM; CDK11B; ACOT8; COQ8A; SURF4; NECAP2; MSH6; MEF2D; DHX57; NDE1; TCEA1; PNKP; TMEM214; FRG1; RPL32; SART3; CDCA5; PSME3; ZNF609; DCTN6; DLG1; CBX5; EEF1A2; CISD1; MNT; NELFCD; TST; KCTD15; TTC37; PRPSAP1; ZNF24; PGK1; PARP14; HK2; PLK1; DST; MLEC; POLA1; COG4; EIF4G3; HNRNPD; CELF1; AP2A2; RXRA; B3GLCT; XRCC5; TMEM97; MORC3; IRF2BP2; SLC25A5; GCN1; QRICH1; CNOT11; UBR5; NOSIP; SUCLG1; KAT7; DPY19L1; EIPR1; ITGAV; TMCO1; TMEM30A; RPS19; FOXK1; SNTB2; QKI; ACAD11; MGA; AP1M1; NKTR; ZNF318; NCOR1; DAD1; PSMC4; RPL13; SUGP2; RDH10; CAMK2D; MACF1; EFL1; DROSHA; CTSB; CTR9; RSBN1; GALK1; CUL3; ACOX1; NIBAN2; ANP32B; FNDC3A; ABCB10; KDM1A; WDR13; DIS3; CSK; PALM; SDHA; HSD17B4; UBXN1; ABCD3; CDH1; ANXA2; TONSL; RMND1; ANXA11 |
| Hypo/Con downregulated DEPs (371) | KRT6B; APOB; NOA1; EPCAM; NDFIP1; NOP53; NDFIP2; NIBAN1; ERAL1; COA4; KIAA1671; SLC38A2; CDK6; GFER; IFNGR1; SLC7A1; ERAP1; NAPRT; PDLIM1; TXNDC17; TAX1BP1; ELP1; MRPL45; TIMM8B; ZPR1; GSDME; MRPL3; CALCOCO2; PDCL3; MRPL41; ADIRF; PFAS; NME2; CDC123; ADGRG1; HSPB1; GTPBP10; HINT1; SQSTM1; COL17A1; COPZ1; KYNU; EIF2B2; PRMT3; SLC4A2; C1QBP; RABGGTB; MRPL50; IMPDH1; KPNA4; HMGA2; KNOP1; MRPS16; USP47; UCHL3; FASTKD2; OLA1; UTP14A; OGA; SLC7A2; MED10; THOP1; ANXA8; KPNA3; NFXL1; CD151; RRS1; LRP8; TCF25; CRYBG1; ATXN10; SRXN1; COA6; DCAF13; COA7; ADSS2; TMEM106B; MED22; ARHGDIA; COX17; GDI2; BET1L; NAP1L1; RRP1; GSTO1; ARHGDIB; XPOT; ATP6V0A2; ABCC2; JPT1; EBNA1BP2; ISG15; OSBPL9; FSCN1; PNPO; NDUFB1; FZD6; CCT2; THEM4; GPATCH4; TMEM59; ENOPH1; WDR46; PEX19; ANXA3; WDR74; CHCHD4; QTRT1; MAPK14; PSMF1; TNFRSF1A; POLR1G; HGH1; PINK1; DDX56; NT5C; MRPS30; ALDH1A3; SLC19A1; URM1; IFIT2; CDC42SE2; MRPL19; ANKRD28; RNF149; PNO1; AATF; COTL1; SARS1; MPHOSPH10; COX6A1; HSPBP1; MRPL4; CSE1L; TIMM13; RAB11A; PM20D2; IFITM3; TM9SF1; BYSL; LCP1; PRDX6; CCT7; DNTTIP2; ACP2; UTP6; COQ8B; PPA1; GSTP1; CAPN2; GNPDA1; GRSF1; SFN; CTPS1; ESD; TMEM40; B3GNT3; ME1; CCDC137; ADH5; MRPL42; DAP3; DPP3; ACTN1; OCLN; DCTPP1; CCT3; SLC20A1; MRPL24; RPS19BP1; ASNS; PSAT1; TIMM10; PPM1A; SNX17; SERPINB5; NEDD4L; SYAP1; SPAG9; ; NIFK; SOWAHC; MRPL55; NOB1; RRM1; PAFAH1B2; FKBP10; LRPPRC; TUBB6; PARN; SQOR; TPM4; IMP3; MRPS34; NME1; MTHFS; MRPL23; CAP1; MRPS18B; CHD7; BOP1; MTND1; SERPINB6; RAB3GAP2; PLS3; NDUFA5; MAK16; PSMG4; HMGCS1; TNFRSF10B; DDX21; NRDC; UTP20; SRM; PPP1R7; TTC1; IMPA1; TNPO1; MTHFD2; RSL1D1; PDCD11; ATG3; ACBD3; PTCD1; DDX10; PPP1R9B; ABHD14B; SLC1A5; FNTA; MRPL11; LTA4H; PGM2; PPAT; RMDN1; GGCT; PNP; CAPNS1; MRPL44; GFUS; ANXA1; ZNF644; ARID2; SRSF2; MRPL49; UBE2H; PUM3; CHTOP; RRP15; STAMBP; IRF6; HINT2; SLK; CHORDC1; MRPS6; EIF6; ITPK1; XPNPEP3; RBM28; ACTN4; EIF1AD; FLVCR1; TFB2M; IPO7; MUC13; YWHAZ; CNDP2; MTERF3; RBM34; LYPLAL1; UBQLN2; THBS1; MRPL38; SRSF5; MRPL1; MRPL28; DDX52; NOL6; IPO4; NSUN4; IPO5; CCT8; SETD2; ITM2B; RRP36; LAS1L; MED31; MRPL39; SDC1; ETHE1; PSMD5; TCP1; PRXL2A; BAZ1A; PPP6R1; NLN; PPIG; UBA5; CYP2S1; GOT1; BRIX1; KARS1; RER1; TMX2; GLRX3; DDX27; COX7A2L; RGPD5,RGPD6; USP3; TUBB4B; MRPL22; IDE; PFDN4; RBM8A; MYBBP1A; FAM126A; ITCH; CCT5; GPC1; FAHD1; RRP7A; MTIF2; MRPL21; ELP2; S100A14; GABARAPL2; BECN1; TM9SF2; FIP1L1; WDR3; KPNB1; SGMS2; HARS1; SNX8; SHTN1; CFL1; NDUFB10; ATL2; UMPS; SLC29A1; EIF2B3; IGBP1; MTPAP; ARCN1; PSPH; GLOD4; TFB1M; GNL3; MRPS7; CCT6A; HEBP2; UTP18; ADGRE5; YWHAB; IGF2R; CDKAL1; CDC27; PBK; VANGL1; ESPN; ADI1; SUMF2; SFPQ; TP63; MRPS2; MRPL9; NOC4L; EEF1D; STX18; IFIT3; STMN1; MRPS23 |
| Hypo+Caf/Hypo upregulated DEPs (383) | KRT6B; MYBBP1A; APOB; PNO1; ITCH; RRP1; MRPL19; ERAL1; NDFIP2; UTP20; RANGAP1; MARCHF5; TIMMDC1; FYTTD1; RPL36AL; UTP25; EMG1; MRPL11; PDCD11; POLR1D; UTP14A; TUBB6; MRPS18B; TMEM106B; ERAP1; SQSTM1; GTPBP10; PRXL2A; MRPL16; CCT5; MRTO4; PUM3; NT5E; RNF149; DDX52; NOA1; DDX21; SQOR; MRPL21; TENT4B; CDK6; ANAPC1; SLC39A1; IFNGR1; TAX1BP1; GNG10; FAM136A; UQCRC1; RBM3; SLC1A5; MRPL9; ZNF622; UTP6; GRSF1; DCAF13; RSL1D1; PNPLA4; CCT7; RMDN1; MTHFD2; DDX18; DIABLO; RBM28; LRPPRC; BOP1; SFN; COPB2; ACP2; WDR74; LAS1L; TFRC; HMGA2; GABARAPL2; IMP3; HARS1; NIFK; TNFRSF10A; LCP1; MPHOSPH10; REEP4; NOP53; SLC4A2; UTP4; CARS1; MAPK14; METTL26; COA6; ELP1; SLC27A2; RRP12; NUDC; RPL7L1; NPM1; PNPO; BRIX1; MRPL37; MRPS18A; COL17A1; CCDC137; SLC39A7; ATP6V0A2; QTRT1; FAM83B; WDR46; DAP3; IGF2R; PHACTR4; HEBP1; ATG3; EEF1D; FASTKD2; WDR36; ADGRG1; ATXN10; FKBP10; KPNA3; UQCRB; TMEM59; CCT3; MTND1; SARS1; CSE1L; MRPL13; UQCRC2; MRPL38; UTP18; DIMT1; TPP2; CALCOCO2; PINK1; IQGAP3; CNIH4; NIBAN1; NEMF; AK2; STX8; VRK2; HMOX1; MTHFS; RRP15; MRPS2; SLC20A1; GSDME; WDR43; RRP7A; IPO4; DDX27; ISG15; CCT6A; EBNA1BP2; GTPBP4; MTERF3; TPM4; UMPS; ESPN; CCT8; PLS3; RBM34; ABCC2; SYAP1; UQCC2; MBD4; CCT4; B3GNT3; ADK; KIAA1671; ASCC1; MTIF2; COTL1; MRPL24; CAPN2; TBL3; MRPL14; COX19; MIEF1; CFL2; DHX33; MRPL23; LRP8; RRS1; ACTN1; KYNU; SH3BGRL3; KANSL2; NDUFA13; ADGRE5; ANAPC7; PCK2; CNDP2; POLE3; GNL3; PSPH; NOLC1; OLA1; SERINC1; COA7; DDX54; MRPL2; KPNA4; MON2; TIMM13; NUDT19; TCP1; COPA; SLIRP; DSTN; TP63; MRPL15; SERPINB6; PES1; THUMPD3; ANAPC5; MRPL39; DMAC1; MRPS34; INTS9; UCHL3; HMGCS1; MIX23; FSCN1; EEF1G; MRPL45; EIF6; GNPDA1; RPF2; NDUFB10; SNRPF; DDX56; HINT2; ALDH1A3; PDLIM1; CIAPIN1; CYP2S1; UCK2; ACBD3; MRPL55; THOP1; NOL6; PFAS; NRDC; BAZ1A; SOWAHC; MRPL43; MTCO1; ADSS2; CTDSPL2; SPATA5; ACTB; NEDD4L; PCF11; MRPL20; PFDN1; RAB3GAP1; WWP2; GFER; TFB1M; FAM126A; ARHGDIB; PFN2; PPA1; NDFIP1; SLC7A1; NME1; NSMCE3; TTC27; TIMM8B; UNC45A; SERPINB5; CTSC; MRPL3; BYSL; GSTP1; SRSF5; HSPBP1; MED6; TXNRD1; DNTTIP2; MYO19; COX17; TTF2; NEDD4; NDUFA4; ANAPC2; PPAT; TPMT; ACTN4; PDCD5; NAPRT; ASNS; PM20D2; TUBB; MRPL4; NIT2; TTC4; IMP4; COPZ1; RGPD5; ARCN1; OSBPL9; NOL7; ANXA3; MRPL50; FPGS; CCT2; MRPL47; BOLA3; EIF2B1; SPAG9; GLG1; PPP3CA; GLRX3; LYPLA1; PARN; RARS2; XPNPEP3; GBF1; COX6A1; NBR1; PTP4A1; GGCT; DPP3; USP15; PTMA; TFB2M; SMG9; RAP2B; BROX; PIK3R4; ESD; AATF; SLC7A2; RPF1; MRPL41; TUBA1C; MRPS6; NOL10; POLR1E; UTP3; MTO1; STEAP3; RCL1; PLEKHH3; EIF2B2; CRYBG1; TACC2; MYO1B; TANC1; THEM4; SNX17; TNFRSF10B; FAM32A; ZDHHC13; SLC7A11; SLC12A6; MRPL1; TSSC4; PEX16; PFN1; HECTD1; OTUB1; THBS1; CD63; MRPS7; GLO1; RPS17; SRM; NSUN5; NME2; WWP1; ZPR1; PRMT3; ABHD14B; URM1; CAPNS1; DDX10; UNC5B; WRN; DPF2; UBE2H; RPS19BP1; PAFAH1B2; RAB3GAP2; UBE2H |
| Hypo+Caf/Hypo downregulated DEPs (304) | KDM3A; ALPI; CTH; STAU1; ISG20; PTGS1; AKR1B1; LAMC2; CLU; SQLE; VIL1; KDM5C; ITGA5; BNIP3; S100A4; PLOD2; MUC1; AURKA; MLF2; RPL30; SLC2A3; NDRG1; LAMB3; UPP1; ARG2; ALDOC; ATP1B1; CHCHD2; BHLHE40; CSRP2; ANLN; AKR1C3; FBLN1; P4HA2; RRAS; NUSAP1; THYN1; MAGED2; WEE1; C1QBP; ALPP; SAMHD1; GRB7; CEBPB; DNAJB6; CMTR1; GTSE1; ANO6; TMBIM6; ALKBH5; STS; TPD52L1; CENPF; KHDC4; SLC25A20; SLBP; F3; TACC3; CHD2; MAFF; MSMO1; ADARB1; ARNT; SSR3; RPP30; PC; COL6A1; CTTNBP2NL; KIFC1; PLEK2; THEM6; IER3IP1; AURKB; NEDD1; IDH2; DDX41; P4HA1; RHPN2; UBE2S; LIMCH1; TPX2; AGR2; CTSB; PCIF1; HELZ2; HLAB; SKP2; MIDEAS; UTRN; COL7A1; ARFGAP3; KIF20A; DRAP1; ANK3; EDF1; H110; ECT2; NFATC2IP; PCSK9; ERO1A; LNPK; INCENP; DEGS1; CHMP2A; MYH10; TMED2; MACROD1; SDF2; MAP1S; SOD2; PBXIP1; CLK1; RPL9; PNPLA6; SLC2A1; SH3GLB1; JUN; SUN2; FBXO2; DYNC1LI2; DDX58; DPP9; TOP2A; KIF2C; SUSD2; C15orf40; JPT2; HDGF; PET100; MPG; GNE; DDX59; LRATD2; PHLDB2; SEC24A; EPHX1; FERMT2; EPS8; GRB10; IRF2BP2; SNX4; CLIP2; DLG1; SMARCAL1; LASP1; DDA1; CDCA8; TRAF2; PALM; KLF5; CDH1; BIN1; CARMIL1; GGH; ANKZF1; BCKDHA; SMARCA2; IWS1; OCRL; ESPL1; MYBL2; SHB; GNPAT; NCOA7; PIEZO1; FAM50A; ARHGAP5; TAPBP; SDC4; PRC1; TLE3; MECP2; NOSIP; SP100; ITSN2; AP1S1; RIOK1; CCNA2; CBX5; TEAD1; RO60; NFKB2; PML; CBX1; SEPTIN6; ARHGAP35; ZNF318; EIF4A2; RPA3; RAPH1; HIF1AN; RBM15B; NCAPD3; MCCC1; RCN1; PDIA5; ANXA2; ATP5ME; GTF2B; ANXA11; BRD8; BDH1; CCNB2; FMR1; IGF2BP2; CDC42BPA; MXRA7; MVP; DTX3L; FNDC3B; SPAG7; TPT1; BTAF1; MORF4L1; CSRP1; BCAM; PGK1; RNGTT; PRKCD; DPYSL2; CDCA5; RRBP1; FAM120B; HAUS6; FTH1; HPCAL1; AGFG1; ULBP2; JUND; PARVB; WNK1; OAS3; TRMT1L; FAM107B; B3GAT3; DUSP3; NKTR; GPD1L; PKP2; TSEN34; SMAD3; MMS22L; MKI67; ERMP1; ALDOA; HMGB2; ATP2B1; MSH6; ANTXR2; ZMYND8; BBX; FAM162A; HLTF; PARP4; CTNNB1; PHF2; PPIL2; IRF2BPL; ZYX; ADD1; USP11; GPS2; BCKDK; RSU1; S100A10; ACOX1; CAMK2G; GPRC5A; NECAP2; KDM3B; SFXN3; ITGB5; MTHFD1; PRDX1; URI1; FOSL2; SRPRA; MBNL2; HNRNPUL2; CIC; SMIM1; SURF1; SELENOT; TMEM30A; EPB41L1; ABCD3; HAGH; P4HB; RPL10A; NAMPT; DDX17; TUBGCP2; ZC3H15; SEC23A; MET; KLC4; NUMB; SEPTIN11; EIF4G3; PEDS1; RPIA; RPL36; AP2A2; ATAD2 |

Supplementary Table 3. The sequences of primers used in this study.

| Gene name | Forward primer | Reverse primer |
| --- | --- | --- |
| NDUFS1 | AAAGGATGTGTTCGAACAACTG | TTCCACCATGACAGACTGAC |
| NDUFS2 | AAGACCTATCTTCAGGCCC | TAGAGAATAGGCCTGTTCGT |
| NDUFS3 | GGATCCTGACAGATTATGGC | CATAACGTAACTCAACATAGCC |
| NDUFS5 | ATTTCGTAGAGTGTTTGCTTCG | TGCTTCCTGATGGTACCTG |
| NDUFS8 | CAGCCACCTACAAGTATGTG | GAACGGGTAGTTGATGGTG |
| NDUFV1 | ATGACTGGAGGCTGAAAGG | ATGTCTTGATCTCGCCCAG |
| NDUFV2 | GGAAGACATGTAAGGAATTTGC | CAGGAGTATCTCTGTGCAC |
| NDUFA5 | AGAGGTGATTCTTCAGGCTG | CTTCCACTAATGGCTCCCA |
| NDUFA8 | GGAGAACTGTCAAAGGTCAC | ATCCGGTCTTGGTCTTGAG |
| NDUFA9 | GTTGTCAACCACCTTGGAC | CGTCCCATTCCAGAAACAG |
| NDUFA11 | ATACACGTTCACTGCAGCT | CCGAGGAAGTAGTTCAGGG |
| NDUFA13 | CTACAAACGGAACTTGCCG | TTCCAATCCCTATGGCCAG |
| NDUFB3 | ATCCAGAAGAAGCTGGCTG | ATCTCCAAGCTTCATTGCG |
| NDUFB8 | CATGTGTATGCAGCTCTTCG | TACTGCTTTGGTCCCACAG |
| NDUFB9 | GATTGCTACAAGGTCCCAG | CTCTTGGCAAAGTAATCAGGA |
| NDUFB10 | CCATCGTCTACATGATGAAAGC | GCTGCCGCTCTATAAATTCTC |
| NDUFB11 | ATGAGAAGAACCCAGACTCC | GACCAGGATGATGGAGACG |
| NDUFC2 | TTGATAACCTAATCCGGCG | TATAGAAGCTGGCGATGCA |
| ISP (UQCRFS1) | ATGTTGTCGGTAGCATCCC | AGAAGCAGGGACATTGAGG |
| QCR7( UQCRB) | ATTAAGAGGGCACTGGACC | CTGGAACAGCAAAGACCTC |
| QCR8( UQCRQ) | CGTCTTCACTAAAGGAATCCC | AAACACTACAAACTGCGGC |
| ATP6 | TCCCTCTACACTTATCATCTTCAC | GACAGCGATTTCTAGGATAGTC |
| ATP8 | TAAATACTACCGTATGGCCCAC | GTGATGAGGAATAGTGTAAGGAG |
